# Extracellular ATP is increased by release of ATP-loaded microparticles triggered by nutrient deprivation

**DOI:** 10.7150/thno.66274

**Published:** 2022-01-01

**Authors:** Valentina Vultaggio-Poma, Simonetta Falzoni, Paola Chiozzi, Alba Clara Sarti, Elena Adinolfi, Anna Lisa Giuliani, Alejandro Sánchez-Melgar, Paola Boldrini, Michele Zanoni, Anna Tesei, Paolo Pinton, Francesco Di Virgilio

**Affiliations:** 1 Department of Medical Sciences, University of Ferrara, Ferrara, Italy.; 2 Department of Inorganic, Organic Chemistry and Biochemistry, Universidad de Castilla-La Mancha, Regional Center of Biomedical Research, Ciudad Real, Spain.; 3 Center of Electronic Microscopy, University of Ferrara, Ferrara, Italy.; 4 Biosciences Laboratory, Istituto Scientifico Romagnolo per lo Studio e la Cura dei Tumori (IRST), IRCCS, Meldola, Italy.

**Keywords:** nutrient deprivation, extracellular ATP, microparticles, tumor microenvironment, P2X7.

## Abstract

**Rationale**: Caloric restriction improves the efficacy of anti-cancer therapy. This effect is largely dependent on the increase of the extracellular ATP concentration in the tumor microenvironment (TME). Pathways for ATP release triggered by nutrient deprivation are largely unknown.

**Methods:** The extracellular ATP (eATP) concentration was *in vivo* measured in the tumor microenvironment of B16F10-inoculated C57Bl/6 mice with the pmeLuc probe. Alternatively, the pmeLuc-TG-mouse was used. Caloric restriction was *in vivo* induced with hydroxycitrate (HC). B16F10 melanoma cells or CT26 colon carcinoma cells were *in vitro* exposed to serum starvation to mimic nutrient deprivation. Energy metabolism was monitored by Seahorse. Microparticle release was measured by ultracentrifugation and by Nanosight.

**Results**: Nutrient deprivation increases eATP release despite the dramatic inhibition of intracellular energy synthesis. Under these conditions oxidative phosphorylation was dramatically impaired, mitochondria fragmented and glycolysis and lactic acid release were enhanced. Nutrient deprivation stimulated a P2X7-dependent release of ATP-loaded, mitochondria-containing, microparticles as well as of naked mitochondria.

**Conclusions**: Nutrient deprivation promotes a striking accumulation of eATP paralleled by a large release of ATP-laden microparticles and of naked mitochondria. This is likely to be a main mechanism driving the accumulation of eATP into the TME.

## Introduction

Caloric restriction reduces tumor incidence and accordingly caloric restriction mimetics (CRM) enhance the anticancer activity of known chemotherapeutics 1. Efficacy of chemotherapy is increased by acute (48 h) starvation, and voluntary fasting increases the response to therapy in cancer patients 2. This is largely due to CRM ability to increase the extracellular ATP (eATP) concentration in the tumor microenvironment (TME), with the outstanding result of reducing the recruitment of immunosuppressive Treg cells 3, 4.

Extracellular ATP acts at plasma membrane P2 receptors 5, among which the P2X7 receptor (P2X7R) subtype is pivotal in inflammation and cancer 4, 6, 7. The P2X7R has a special relationship with ATP because it is both a main sensor of the eATP concentration in the TME 6 and a conduit for eATP release 8. Furthermore, P2X7R stimulation promotes intracellular ATP (iATP) synthesis 9.

Accumulation of eATP in the TME might support tumor progression or on the contrary promote an anti-tumor immune response 4, 6, 10, 11. However, although the pmeLuc bioluminescent probe now allows accurate measurements of eATP in the TME 12, 13, and despite indirect evidence that eATP modulates immune cell recruitment 14, mechanisms involved in eATP accumulation and relationship with cancer cell metabolism are unknown. This is of burning urgency since there is now clear evidence that not only eATP accumulates in the TME but also shapes the activity of leukemia cell precursors in the bone marrow 15, and may be exploited to confer target selectivity to anti-cancer therapy 14, 16, 17. Thus, eATP is now acknowledged as a key determinant of tumor-host interaction 4. In light of future cancer therapies, these unsolved questions make it difficult to design a rationale strategy for its manipulation. Thus, in the present study we set to investigate the relationship between intra- and extra-cellular ATP (iATP versus eATP), P2X7R expression, energy metabolism and microparticle release in the B16F10 melanoma and in the CT26 colon carcinoma cell lines.

We induced nutrient deprivation *in vivo* by administration of the CRM hydroxycitrate (HC), and mimicked the *in vivo* CRM activity by *in vitro* exposing cancer cells to nutrient deprivation (serum starvation). Nutrient deprivation, despite the severe impairment of mitochondrial metabolism and the dramatically reduced iATP levels, caused a large increase in *in vivo* and *in vitro* eATP. The eATP increase was largely due to the P2X7R-dependent accumulation of ATP-laden, mitochondria-containing, microparticles and of naked mitochondria. In conclusion, we describe a mechanism by which nutrient deprivation molds melanoma cell energy metabolism and generates an eATP rich TME.

## Methods

### Reagents

Potassium hydroxycitrate (HC, cat# 59847), adenosine 5'-triphosphate (ATP, cat# A26209), benzoyl ATP (BzATP, cat# B6396), bafilomycin A1 (cat# B1793) and ethidium bromide (cat# E1510) were purchased from Sigma-Aldrich (St. Louis, MO, USA). Tetramethylrhodamine methyl ester (TMRM, cat# T668), MitoTracker Green (cat# M7514) and MitoTracker Red (cat# M22425) (all from Molecular Probes, Leiden, The Netherlands) were dissolved in DMSO to obtain 10 mM (TMRM) or 1 mM (Mitotracker Green and Red) stock solutions, respectively, and then diluted in the appropriate buffer. Carbonyl cyanide a-[3-(2-benzothiazolyl)6-[2-[2-[bis(carboxymethyl) amino]-5-methylphenoxy]-2-oxo-2H-1-benzopyran-7yl]-b-(carboxymethyl)-tetrapotassium salt (FCCP, cat# C2920, Sigma-Aldrich) was solubilized in ethanol to a final stock concentration of 10 mM. For Seahorse analysis, a Seahorse XF Cell Mito Stress Test Kit was used including the following compounds: oligomycin (stock solution 100 µM), FCCP (stock solution 100 µM), and a mix of rotenone/antimycin A (stock solution 50 µM) (cat# 103015-100, Agilent, Santa Clara, CA, USA). Crystal violet was purchased from Sigma-Aldrich (cat# C0775) and used as a 0.1% solution in 10% ethanol.

### Cell culture and transfections

B16F10 melanoma and CT26 colon carcinoma cells were grown in RPMI-1640 medium (cat# R6504, Sigma-Aldrich) as previously described 18. For microparticle release and analysis, cells were cultured in RPMI medium containing 10% extracellular vesicle (EV)-depleted FBS (cat# A2720801, Gibco, Thermo Fisher Scientific, Waltham, MA, USA). B16F10 cell clones stably expressing pmeLuc cells were obtained as previously described 13.To silence the P2X7R, B16F10 cells were transfected with the P2X7 short hairpin RNA (sh-RNA) in pSuper.neo.green fluorescent protein (GFP) vector, a kind gift of Dr Miguel Diaz-Hernandez (Universidad Complutense, Madrid, Spain). Single cell-derived clones (B16F10 P2X7R-shRNA) were obtained by limiting dilution 19.

### Mice strains, tumor generation, *in vivo* imaging and drug administration

The pmeLuc-TG-mouse was generated in collaboration with The Danish Center for Genetically Modified Mice (Aarhus University) as previously described 20. C57Bl/6 mice were purchased from Envigo RMS Srl Bresso, Italy. A total of 2.5 × 10^5^ B16F10-pmeLuc cells were subcutaneously injected into C57Bl/6 wt mice (4-6 week-old male mice, n = 12). On the contrary, pmeLuc-TG-mice (4-6 week-old male mice, n = 12) were inoculated with 2.5 × 10^5^ B16F10 wt cells (not transfected with the pmeLUC plasmid). Tumor size was measured with a calliper, and volume calculated as previously described 18. Luminescence emission was measured at post-inoculum (p.i.) day 5, 7 and 9 (at p.i. d 11 in the pmeLuc-TG-mice) with a total body luminometer (IVIS Lumina, Perkin Elmer, Hopkinton, MA, USA). Mice anesthetized with 2.5% isofluorane were intra-peritoneum injected with 150 mg/kg D-luciferin (cat# E1601, Promega, Madison, WI, USA) and, after a 15 min interval to allow biodistribution, luminescence was captured from dorsal view. Photon emission was quantified using the Living Image® software (Perkin Elmer) and averaged as photons/seconds/cm^2^/steradian (abbreviated as p/s/cm^2^/s). Potassium hydroxycitrate (300 mg/kg) or vehicle (sterile phosphate-buffered saline, PBS) were i.p. injected (100 μL) every 2 days starting at p.i. d 5, i.e. at first tumor mass detection.

### *In vitro* measure of ATP levels

ATP was measured with the ENLITEN rLuciferase/Luciferin reagent (cat# FF2021, Promega, Milan, Italy) with a Perkin Elmer Wallac Victor3 1420 luminometer (Perkin Elmer, Wellesley, Massachusetts, USA). Briefly, to measure eATP, 5 × 10^3^ B16F10 cells/well were plated in ninety-six-well microplates (cat# 655077, Greiner Bio-One). Following adhesion, cells were incubated for 48 h in the presence or absence of serum, or alternatively treated with 1 mM HC in serum-containing medium. Then, supernatants were removed, and 50 μL of diluent buffer (FireZyme, San Diego, CA, USA) to stabilize ATP, and 50 μL of ENLITEN reagent were added to each well. To measure total iATP content, cells were lysed with 5 μL of milli-Q water and supplemented with 45 μL of diluent buffer (FireZyme) and 50 μL of ENLITEN reagent. Extracellular ATP was also measured with the pmeLuc probe in the IVIS Lumina luminometer. 50 × 10^3^ B16F10-pmeLuc cells/well were plated in 24-well plate and cultured in the presence or absence of serum, or treated with HC (1 mM) in serum-containing medium. D-Luciferin (Promega) was added to each well at a concentration of 300 μg/mL. Luminescence is expressed as total photons measured during a 5 min acquisition.

### Cell proliferation assay and lactate dehydrogenase (LDH) measurement

Cell proliferation in the presence or absence of HC was measured with crystal violet as described previously 9. Lactate dehydrogenase release was measured as described previously 21.

### Cytosolic calcium concentration measurement

Cytosolic Ca^2+^ was measured with the fluorescent Ca^2+^ indicator Fura-2-acetoxymethyl ester (Fura-2/AM) (cat# F1221, Thermo Fisher Scientific) as described previously 22.

### Seahorse measurement of mitochondrial respiratory flux and glycolysis

Oxygen consumption rate (OCR) and extracellular acidification rate (ECAR) were measured with the Seahorse Bioscience XF96 Extracellular Flux Analyzer (Seahorse Bioscience, Agilent) as previously described 9. Cells were seeded in triplicate at different time points in XF96 96-well cell culture plates (Seahorse Bioscience, Agilent) in a volume of 100 µL in complete RPMI-1640 medium at the density of 5 × 10^3^/well (for the 48 h incubation) or 6 × 10^3^/well (for the 24 h incubation).

### Lactate measurement

Lactate release was measured with the Lactate Colorimetric Assay Kit (cat# ab65331, Abcam, Cambridge, UK), according to manufacturer's indication. Briefly, a total of 10 × 10^3^ cells/well were plated in 24-well plate in the presence or absence of serum and processed as described previously 23.

### Mitochondrial membrane potential measurement

Mitochondrial membrane potential (ΔѰm) was measured with 50 nM tetramethyl rhodamine methyl ester (TMRM) with a confocal microscope (LSM 510, Carl Zeiss, Oberkochen, Germany) equipped with a plan-apochromat ×63 oil immersion objective as previously described 24.

### Mitochondria staining

Cells were plated onto 40-mm glass coverslips at a density of 6 × 10^3^/coverslip, cultured in the presence or absence of serum for 24 h or 48 h, stained for 15 min at 37 °C with the mitochondria-selective dye Mitotracker Green FM (200 nM) in RPMI-1640 medium without phenol red and serum, and analyzed with an inverted Nikon Eclipse TE300 microscope equipped with a 100×/05 -1.3 NA oil Iris objective (Nikon, Tokyo, Japan). Coverslips were then mounted in a thermostated Leyden Chamber (37 °C; Medical Systems, Greenvale, NY, USA) and placed on the microscope stage. Images were captured with a back-illuminated charge coupled device (CCD) camera (Princeton Instruments, Trenton, NJ, USA) using the Metamorph software (Universal Imaging, West Chester, PA, USA).

### Microparticle purification

For characterization of cell-derived microparticles, B16F10 cells were seeded in ten T175 flasks at a concentration of 2 × 10^6^/flask (for the 24 h incubation), or 1 × 10^6^/flask (for the 48 h incubation). To avoid interference by extracellular vesicles (EV) carried with serum 25, cells were supplemented with microvesicle-free serum and serum-free medium. At each given time point, supernatants were harvested and centrifuged at 3000 g for 20 min at 4 °C to get rid of floating cells and debris. Microparticles were then purified by centrifuging supernatants at 100,000 g in a Beckman L8-M Ultracentrifuge equipped with a 70Ti rotor (Beckman Coulter SpA, Milano, Italy) for 90 min at 4 °C. Microparticle pellets were collected and resuspended in 70 µL of appropriate buffer.

### Nanoparticle Tracking Analysis (NTA)

Nanoparticle tracking analysis was applied to measure particle size and concentration. Purified microparticles were diluted in 70 µL of 0.22 µm-filtered PBS to a final volume of 1 mL. Samples were loaded into the sample chamber of a NanoSight LM10 instrument (Malvern Panalytical, Malvern, UK), and optimal microparticle concentration was determined by pre-testing the ideal particle per frame value (20-100 particles/frame). Camera level was increased until all particles were distinctly visible and the ideal detection threshold was determined to include as many particles as possible. Five videos of 60 seconds each were recorded for each sample at a constant temperature of 25 °C and syringe speed of 40 µL/s. After capture, videos were analyzed with the in-build NanoSight Software NTA 3.1 Build 3.1.46.

### Measurement of microparticle-associated mitochondrial fluorescence

B16F10 cells at a density of 1 × 10^6^/flask were incubated in RPMI-1640 serum free medium in the presence of Mitotracker Green (200 nM) or Mitotracker Red (300 nM) for 20 min at 37 °C, rinsed and then incubated in RPMI-1640 medium in the presence or absence of EV-depleted serum for 24 and 48 h. Released microparticles were resuspended in 50 µL of Ca^2+^-containing sucrose medium (300 mM Sucrose, 1 mM K_2_HP0_4_, 1 mM MgSO_4_, 5.5 mM D-Glucose, 1 mM CaCl_2_, 20 mM Hepes) and fluorescence measured with a Cary Eclipse Fluorescence Spectophotometer (Agilent Technologies) at 480 nm excitation and at 516 nm emission wavelengths, and 581 nm excitation and 644 nm emission wavelengths for Mitotracker Green and Mitotracker Red, respectively.

### Measurement of ATP content of released microparticles

Microparticles were purified from B16F10 or CT26 cells as described above and the ATP content measured with the ENLITEN rLuciferase/Luciferin reagent. Luminescence was converted to the ATP concentration with an independent calibration and normalized on the microparticle protein concentration.

### Electron microscopy and immunogold labeling of isolated microparticles

Microparticles were processed as described in 9. Ultrathin sections were prepared with the Reichert Ultracut S Ultramicrotome (Leica Biosystems, Milan, Italy) and analyzed with a EM910 Zeiss transmission electron microscope (Carl Zeiss). For immunogold labeling, isolated microparticles were fixed in 2% paraformaldehyde for 1 h at room temperature and then treated with 0.05% Triton X100 for 20 min at room temperature. After permeabilization, non-specific binding site were blocked with 2% BSA/PBS 20 min at room temperature, and the microparticles were then incubated overnight with the primary rabbit anti-TOM20 antibody (Sigma-Aldrich, cat# HPA011562) at 1:50 dilution in 0.2% BSA/PBS at 4 °C. After washing with PBS, microparticles were incubated for 1 h at room temperature with 20 nm Protein A-conjugated colloidal gold particles (Sigma-Aldrich, cat# P6855) diluted 1:50 in 0.2% BSA/PBS. Finally microparticles were rinsed in PBS, fixed with 2.5% glutaraldehyde and processed for electron microscopy.

### Western blot analysis

Cell lysates or isolated microparticles were solubilized in RIPA-buffer (150 mM NaCl/0.1% SDS/0.5% Na-deoxycholate/1% Triton X-100/50 mM Tris, pH 7.2) and Western blot carried out as described 9. The following antibodies at the specified dilutions were used: rabbit anti-P2X7R (Merck-Millipore, cat# AB5246), diluted 1:500 in TBS-t buffer (TBS plus 0.1% Tween 20) and 2.5% milk; rabbit anti-non-muscle myosin IIA antibody (Abcam, cat# ab75590), diluted 1:1000 in TBS-t buffer and 2.5% BSA; rabbit anti-P-AMPK (Thr172) (Cell Signaling, cat# 2535) and anti-AMPKα (Sigma-Aldrich, cat# SAB4502329), both diluted 1:1000 in TBS-t buffer and 5% BSA; rabbit anti-LC3B (Sigma-Aldrich, cat# L7543), diluted 1:2000 in TBS-t buffer and 5% BSA; rabbit anti-TOM20 (Sigma-Aldrich, cat# HPA011562), mouse anti-TIM23 (BD Bioscience, cat# 611223) and rabbit anti-carbonic anhydrase II (CA2) (Sigma-Aldrich, cat# SAB 2900749), diluted 1:1000 in TBS-t buffer and 5% milk; rabbit anti-HSP60 (Sigma-Aldrich, cat# SAB4501464), diluted 1:2000 in TBS-t buffer and 5% milk; rodent anti-OXPHOS (Abcam, cat# ab110413), diluted 1:1000 in PBS 1% milk; rabbit anti-Flotillin-1 (D2V7J) (Cell Signaling, cat# 18634) and mouse anti-Alix (3A9) (Cell Signaling, cat# 2171), both diluted 1:1000 in TBS-t buffer and 5% BSA; rabbit anti-Calnexin antibody (GeneTex, cat# GTX109669), diluted 1:5000 in TBS-t buffer containing 5% BSA. Membranes were incubated with secondary goat anti-rabbit (cat# 31460, Invitrogen, Termo Fisher Scientific) or anti-mouse (cat# 62-6520, Invitrogen, Termo Fisher Scientific) HRP-conjugated antibodies at a 1:3000 dilution for 1 h at room temperature. Detection was performed with ECL reagent (cat# WP20005, Invitrogen, Thermo Fisher Scientific) in a LI-COR blot scanner (LI-COR Biosciences, Lincoln, NE, USA). Densitometry was performed with ImageJ software (public domain available at http://rsb.info.nih.gov/nih-image/).

### Cell isolation from tumor tissue

Tumors grown in pmeLuc-TG-mice were excised, cut in small pieces and resuspended in RPMI-1640 containing 1.5 mg/mL Type I Collagenase (cat# SCR103, Sigma-Aldrich), 100 mg/mL DNase I (cat# 04716728001, Roche), in the absence of FBS, and digested for 40 min at 37 °C under gentle agitation. The digestion product was then filtered through a 40 μm cell strainer to obtain a single-cell suspension. Infiltrating inflammatory cells were then enriched by Percoll (Sigma-Aldrich, cat # P1644) density gradient following the manufacturer's protocol.

### Real-time quantitative RT-PCR

Total RNA was extracted using TRIzol Reagent (cat# 15596026, Thermo Fisher Scientific) and the PureLink RNA Mini Kit (cat# 12183018A, Invitrogen). For pmeLuc analysis, RNA was extracted from spleens from P2X7R wt mice (as a negative control), from spleens from pmeLuc-TG-mice (as a positive control) and from cells eluted from tumors growing in pmeLuc-TG-mice. For P2X7R analysis, total RNA was extracted from 2 × 10^6^ B16F10 wild-type cells cultured in presence or absence of serum at different time points and transcribed using the High Capacity cDNA Reverse Transcription Kit (cat# 4374966, Applied Biosystems, Thermo Fisher Scientific) as described previously 26. For pmeLuc analysis, iTaq Universal SYBR Green Supermix (cat# 1725120, Bio-Rad Laboratories, Hercules, CA, USA) and the following primers were used: forward 5' CCAGGGATTTCAGTCGATGT 3', reverse 5' AATCTCACGCAGGCAGTTCT 3'; for G3PDH, forward 5' GTTGTCTCCTGCGACTTCAAC 3', reverse 5' TGCTGTAGCCGTATTCATTGTC 3' (final concentration 20 pM each). For P2X7R, TaqMan Gene Expression Master Mix (cat# 4369016, Applied Biosystems) and customized primers were used: P2X7R (Mm00440578; Applied Biosystems), mouse GAPD (GAPDH) endogenous control (VIC®/MGB probe, primer limited) (cat# 4352339E, Applied Biosystems). Real Time PCR was carried out in the AB StepOne Real Time PCR (Applied Biosystems), and all results were analyzed with the 2^-Δ/ΔCT^ method.

### Statistical analysis

Data were analyzed with the GraphPad Prism 6 software (GraphPad Software, Inc., La Jolla, CA, USA). Statistical significance was calculated with a two-tailed Student's t test assuming equal SD and variance. All data are shown as mean ± standard error of the mean (SEM). Differences were considered significant at P < 0.05. Coding: *P < 0.05; **P < 0.01; ***P < 0.001; ****P < 0.0001.

## Results

### Caloric restriction caused eATP release and inhibition of tumor cell growth

**Figure 1:**
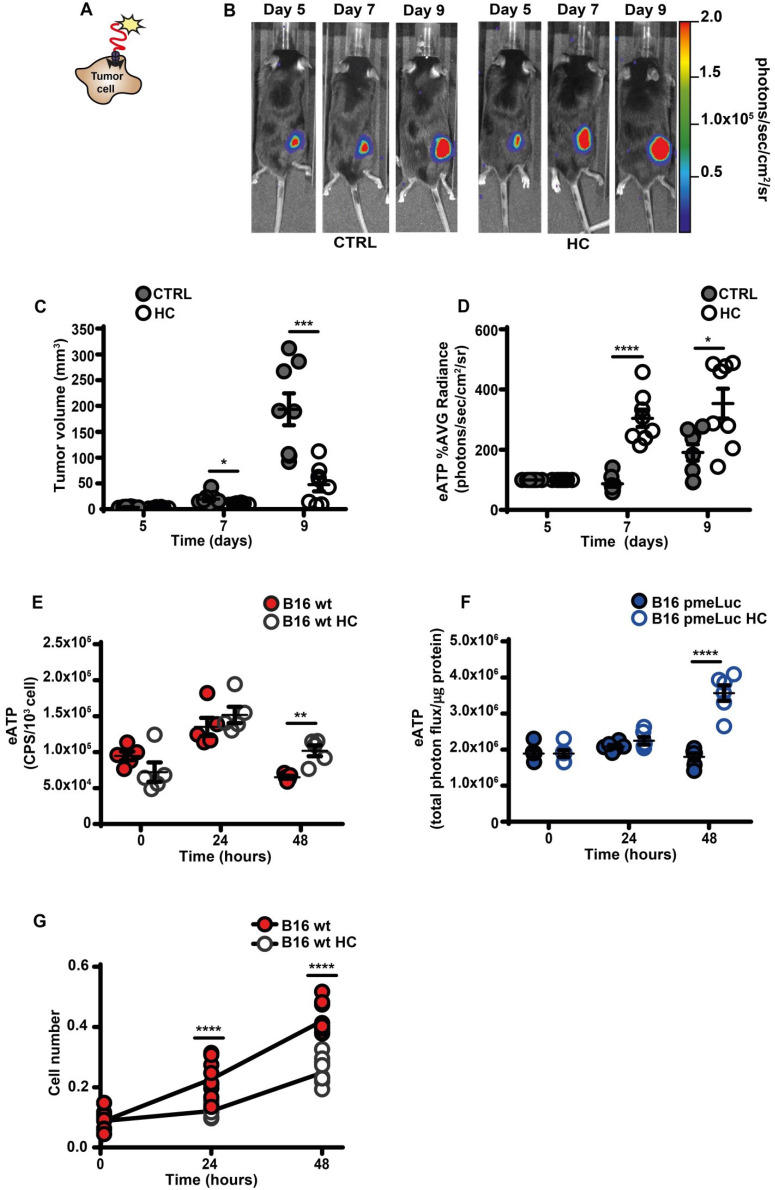
** Hydroxycitrate promotes ATP release from B16F10 melanoma cells and growth inhibition *in vivo* and *in vitro.*** (**A**) Schematic rendition of pmeLUC-transfected B16F10 (B16F10-pmeLUC) cells showing plasma membrane localization of the pmeLUC probe. (**B**) Representative images showing luminescence emission from control (left) and hydroxycitrate (HC)-treated (right) C57Bl/6 wt mice (n = 8) inoculated with B16F10-pmeLUC cells (2.5 × 10^5^) into the right hind flank; HC, 300 mg/kg, was i.p. administered at p.i. d 5, 7 and 9. (**C**) Volume of tumors from control (closed circles) and HC-treated (open circles) mice assessed *in vivo* by calliper at the indicated time points. (**D**) *In vivo* extracellular ATP (eATP) levels in tumor-bearing mice estimated by pmeLUC luminescence average (AVG) emission (p/s/cm^2^/sr). Data are reported as percentage luminescence increase over d 5. (**E**) *In vitro* eATP levels measured at the indicated time points with soluble luciferase in the supernatant of B16F10 cells (5 × 10^3^) incubated in RPMI-1640 medium in the absence (closed red circles) or presence (open circles) of 1 mM HC. To measure ongoing eATP release rather than eATP accumulation into the cell supernatant, the incubation medium was withdrawn right before eATP measurement, and 50 μL of buffer solution supplemented with 50 μL of rLuciferase/Luciferin reagent were added to each well. CPS (counts per second) were normalized to cell content measured with crystal violet (n = 5). (**F**) *In vitro* eATP levels measured at the indicated time points in B16F10-pmeLUC monolayers (50 × 10^3^) incubated in RPMI-1640 medium in the absence (closed blue circles) or presence (open circles) of 1 mM HC. Total photon flux was normalized to cell protein (µg) (n = 6). (**G**) *In vitro* proliferation of B16F10 cells (3 × 10^3^) incubated in RPMI-1640 medium in the absence (closed red circles) or presence (open circles) of 1 mM HC. Cell number was measured with crystal violet (n = 10). Data are shown as mean ± SEM. *P < 0.05; **P < 0.01; ***P < 0.001; ****P < 0.0001.

An increased eATP concentration is a feature of the TME 4, 12, 13, 27. This discovery prompted the development of highly innovative and tumor-selective therapies 14, 16, 28. Accumulation of eATP in tumors recruits immune cells, activates autophagy, enhances tumor antigen presentation and potentiate the anti-tumor response 3, 29. We used B16F10 mouse melanoma cells stably transfected with the eATP-selective pmeLuc probe to show that *in vivo* administration of the CRM HC nearly abrogated tumor growth and almost doubled TME eATP (Figure 1A-D). Hydroxycitrate also increased *in vitro* eATP levels, whether measured with the usual luciferase/luciferin soluble assay (Figure 1E), or with pmeLuc (Figure 1F), and inhibited cell growth (Figure 1G).

**Figure 2:**
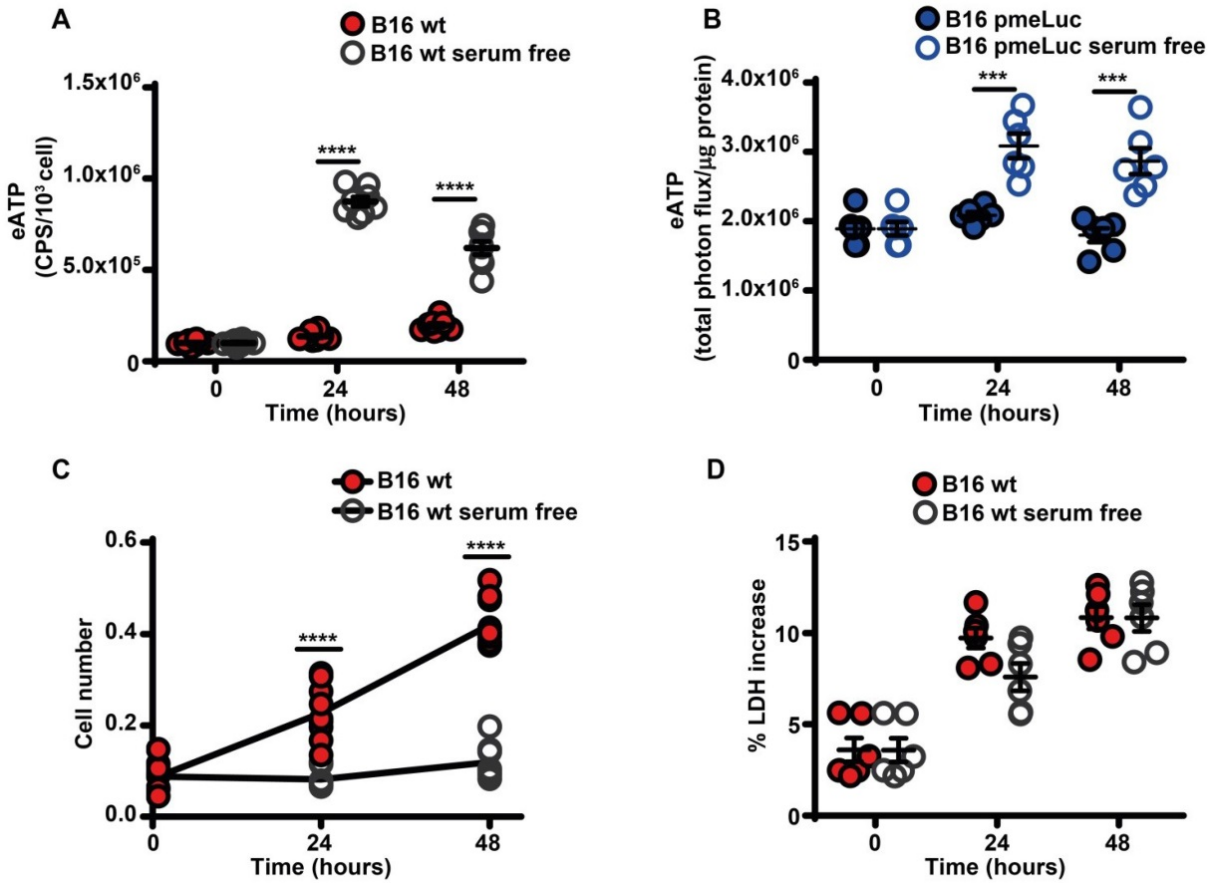
** Serum deprivation causes ATP release and cell growth inhibition**. (**A**) Extracellular ATP levels in B16F10 cells (5 × 10^3^) cultured in RPMI-1640 medium in the presence (closed red circles) or absence (open circles) of serum. eATP was measured with soluble luciferase. CPS (counts per second) were normalized to cell content measured with crystal violet (n = 8). (**B**) eATP levels measured in B16F10-pmeLUC monolayers (50 × 10^3^) incubated in RPMI-1640 medium in the presence (closed blue circles) or absence (open circles) of serum. Total flux was normalized to cell protein (µg) (n = 6). (**C**) *In vitro* proliferation of B16F10 cells (3 × 10^3^) incubated in RPMI-1640 medium in the presence (closed red circles) or absence (open circles) of serum (n = 10). (**D**) Lactate dehydrogenase (LDH) release from B16F10 cells (10 × 10^3^) incubated in RMPI-1640 medium in the presence (closed red circles) or absence (open circles) of serum. LDH release is shown as percentage release of total LDH cell content (n = 6). Data are shown as mean ± SEM. ***P < 0.001; ****P < 0.0001.

Serum starvation increased eATP release from B16F10 cells (Figure 2A-B), and inhibited cell proliferation (Figure 2C). The large release of eATP in the absence of serum was not due to cell death as no increased release of the cytosolic enzyme lactic dehydrogenase was detected at any time (Figure 2D).

Another tumor cell line, the CT26 colon carcinoma cells, also responded to serum starvation or HC supplementation with an increased release of eATP (Figure S1).

Serum is known to contain ecto-ATPases that might hydrolyze eATP, thus the large difference in eATP release from serum-starved versus serum-supplemented cells could be at least in part due to enhanced eATP hydrolysis by soluble ecto-ATPases carried in the serum. As shown in Figure S2A, hydrolysis of eATP was strongly accelerated by the presence of serum in the absence of cells, however in the presence of cells rate of eATP hydrolysis was not affected by the presence or absence of serum, indicating that in cell monolayers the main determinant of ATP hydrolysis is cell-associated ectonucleotidase activity (Figure S2B).

A major transducer of signals conveyed by eATP and a potent stimulant of proliferation of B16F10 melanoma cells is the P2X7R 18, 30. Thus, we verified whether lower proliferation in serum-starved melanoma cells was associated with reduced P2X7R function. Serum starvation significantly reduced resting (Figure S3A) and BzATP-stimulated (Figure S3B) cytosolic Ca^2+^ levels ([Ca^2+^]_i_), with a negligible effect on P2X7R mRNA and no effect on protein levels (Figure S3C-E).

### Serum starvation inhibits oxidative phosphorylation and decreases intracellular ATP

**Figure 3:**
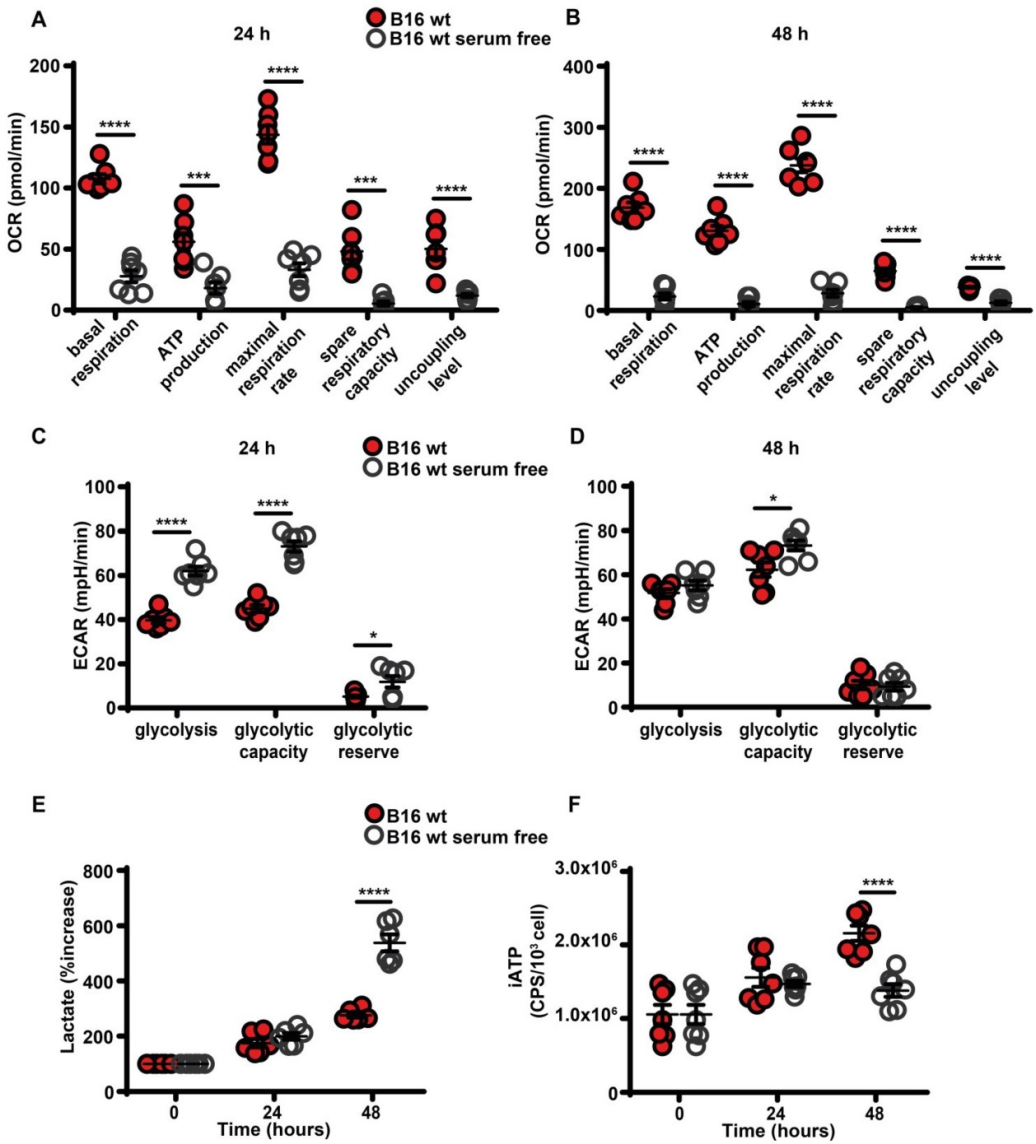
** Serum deprivation impairs mitochondrial energy metabolism and reduces intracellular ATP levels**. B16F10 cells (5-6 × 10^3^) were incubated in RPMI-1640 medium in the presence (closed red circles) or absence (open white circles) of serum for 24 (**A** and** C**) or 48 (**B** and **D**) h, then oxygen consumption rate (OCR) (**A** and **B**) and extracellular acidification rate (ECAR) (**C** and **D**) were measured in a Seahorse Analyzer (n = 7) (mpH= milli pH units). (**E**) B16F10 cells (10 × 10^3^) were incubated in the presence (closed red circles) or absence (open white circles) of serum for the indicated time and lactate release was measured in the cell supernatants. Lactate release is expressed as percent increase over time zero. (**F**) B16F10 cells (5 × 10^3^) were incubated in RPMI-1640 medium in the presence (closed red circles) or absence (open white circles) of serum, at the indicated time were lysed and intracellular ATP (iATP) measured by luciferase assay. CPS (counts per second) were normalized to cell content determined by crystal violet (n = 7). Data are shown as mean ± SEM. *P < 0.05; ***P < 0.001; ****P < 0.0001.

Maintenance of high eATP levels by starved cells is counterintuitive as nutrient deprivation should reduce intracellular energy synthesis, thus decreasing iATP. Seahorse analysis showed that in the absence of serum all respiratory indexes were severely reduced (Figure 3A-B), and in parallel glycolytic activity and lactate release were increased (Figure 3C-E). Accordingly, iATP levels were reduced, especially after 48 h of incubation in the absence of serum (Figure 3F).

**Figure 4:**
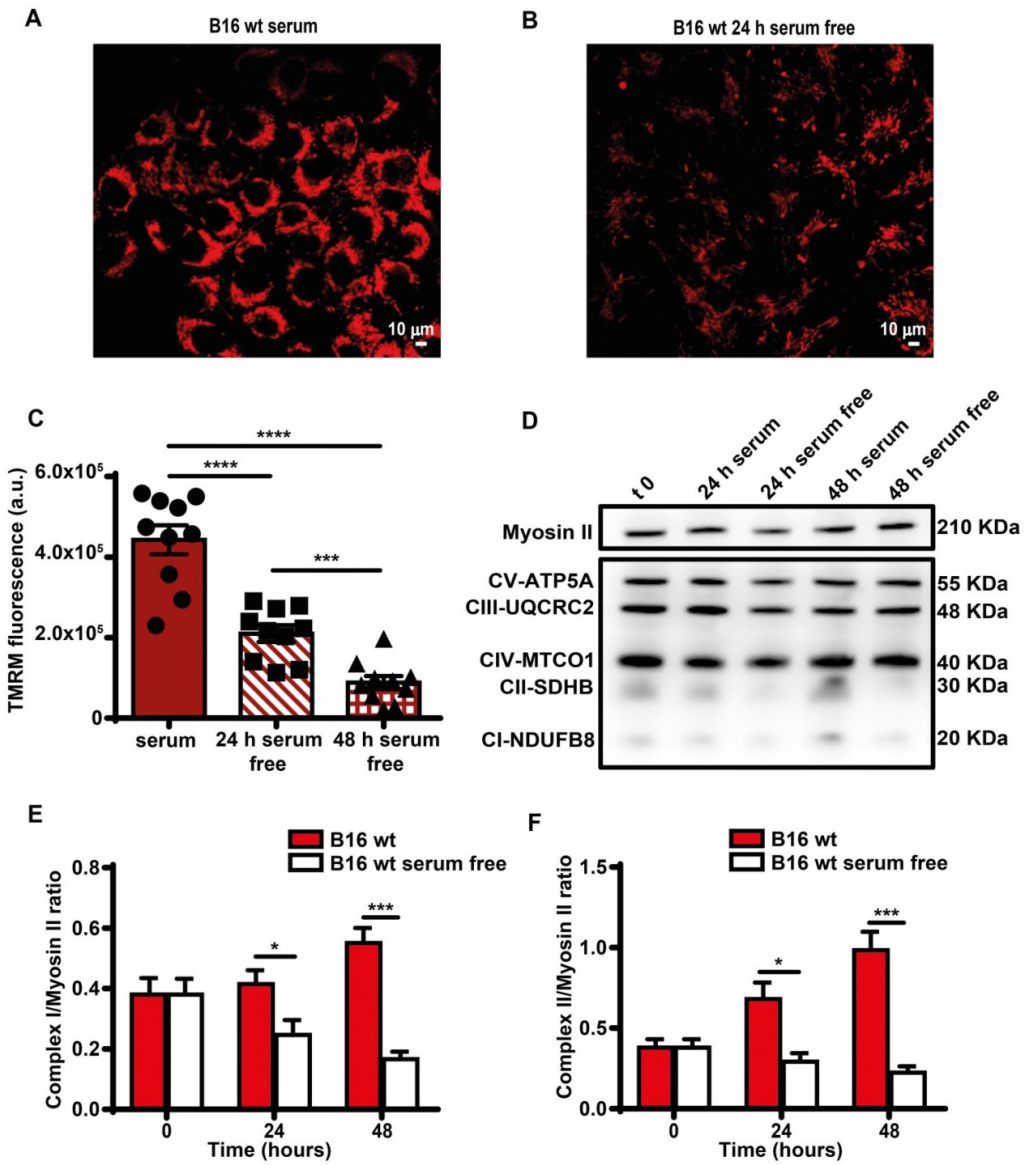
** Serum deprivation reduces mitochondrial membrane potential and Complex I and II levels**. B16F10 monolayers were incubated in the presence (**A**) or absence (**B**) of serum for 24 h, then rinsed twice with warm PBS to remove serum and cell debris, and loaded with tetramethyl rhodamine methyl ester (TMRM, 50 nM) in saline solution (see Methods). Fluorescence was measured with a Zeiss LS510 confocal microscope at an emission wavelength of 570 nm using red laser excitation (543 nm). Images were analyzed with ImageJ software. Bars = 10 µm. (**C**) Mitochondrial membrane potential (Ѱm) expressed as ratio between TMRM fluorescence (in arbitrary units, a.u.) before and after FCCP addition (n = 10). (**D**) Western blot analysis of respiratory chain complexes from B16F10 serum-supplemented and serum-starved cells at t 0, 24 and 48 h. Fifteen micrograms of protein were loaded in each lane. Densitometry of Western blot analysis of mitochondrial Complex I (**E**) and Complex II (**F**) at indicated time points (n = 4). Data are shown as mean ± SEM. *P < 0.05; ***P < 0.001; ****P < 0.0001.

Direct measurement showed a smaller decline of iATP than estimated by Seahorse, likely because glycolysis partially compensated the respiratory deficit. Mitochondria were largely depolarized (Figure 4A-C), and expression of respiratory chain Complexes I and II decreased (Figure 4D-F). Functional changes were paralleled by a striking fragmentation of the mitochondrial network (Figure S4).

Nutrient deprivation increases autophagy and mitophagy that might be responsible for mitochondrial fragmentation. However we were unable to detect an increase in either responses (Figure S5A-C). Likewise, we were unable to detect any increase in AMPK activation (Figure S5D), a pathway known to be stimulated by nutrient depletion 31.

Several pathways known to be present in melanoma cells support non-lytic ATP release: pannexin-1 32, 33, connexin-43 34, 35, and the P2X7R itself 8, 18. However, B16F10 cells also actively release exosomes/microvesicles (microparticles) 36, 37, thus we set to investigate whether shed microparticles might be responsible for eATP release.

### Serum starvation promotes release of free mitochondria and of ATP-laden microparticles

**Figure 5:**
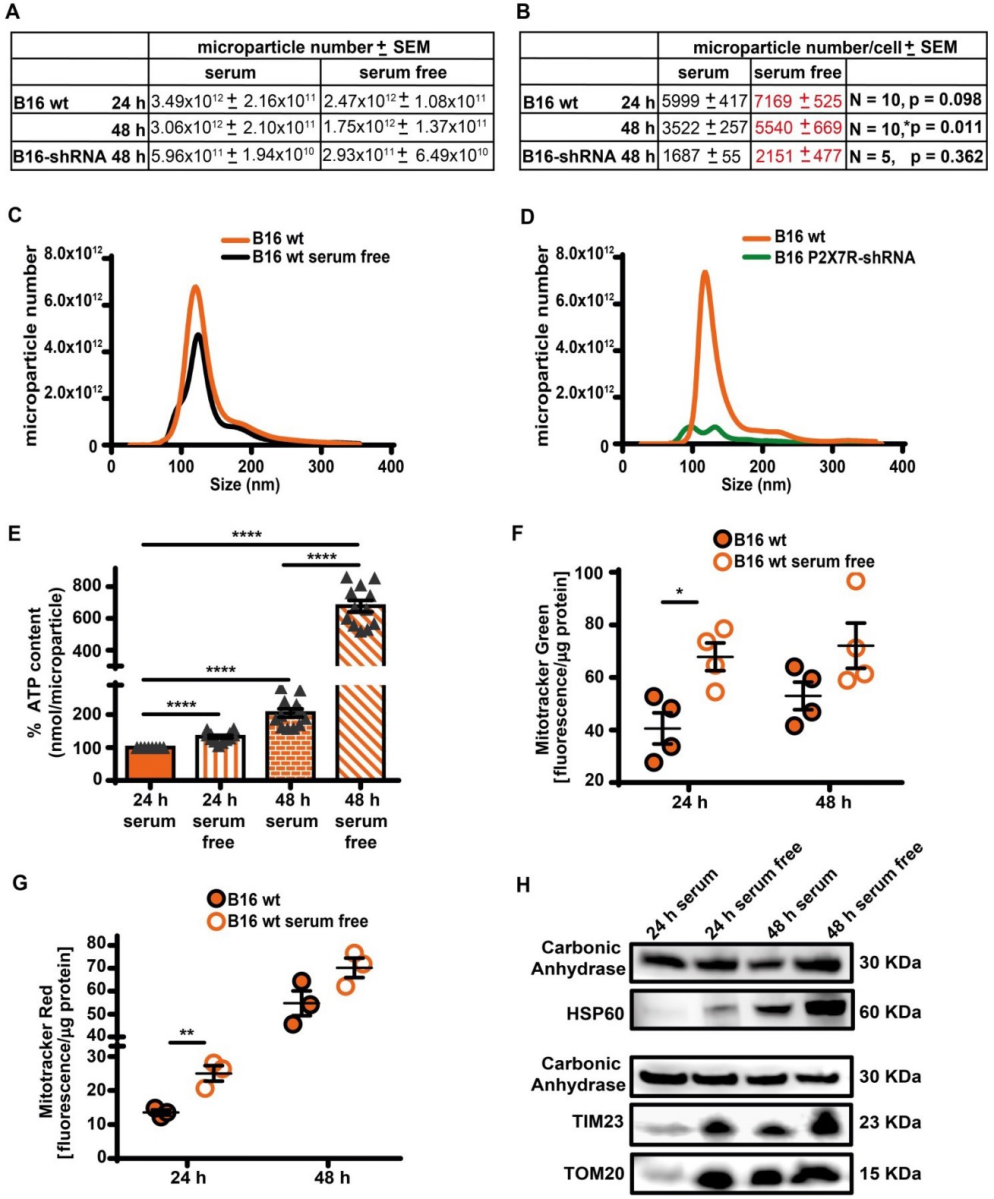
** Serum deprivation enhances microparticle release in a P2X7R-dependent fashion**. (**A**) Control (B16 wt) or P2X7R-silenced (B16-shRNA) B16F10 cells were incubated at a concentration of 1 × 10^6^ (for the 48 h incubation) or 2 × 10^6^ (for the 24 h incubation)/flask in the presence or absence of serum depleted of extracellular vesicles (EV) for the indicated time. At the end of this incubation, cells were counted again, the supernatant removed and extracellular particles isolated as described in Methods. Microparticles were measured with the NanoSight system as described in Methods. Total microparticle number is reported in panel (**A**), while particle number normalized to cell number is reported in panel (**B**). Representative traces of size distribution of microparticles released from B16F10 wt cells incubated in the presence or absence of serum (**C**), and from wt or P2X7R-silenced B16F10 cells (**D**). (**E**) ATP content of microparticles isolated from B16F10 cells incubated in the presence or absence of serum. Microparticles were isolated as described in (**A**). Data are reported as percent increase over ATP content at 24 h in the presence of serum (n = 12). (**F-G**) B16F10 cells (1 × 10^6^/flask) were loaded with Mitotracker Green (200 nM, n = 4) (**F**) or Mitotracker Red (300 nM, n = 3) (**G**) in RPMI-1640 serum free medium, rinsed twice with warm PBS and then incubated in RPMI-1640 medium in the presence (closed orange circles) or absence (open white circles) of EV-depleted serum for 24 and 48 h. Microparticles were isolated and fluorescence measured as described in Methods. Fluorescence emission was normalized over microparticle protein. Data are shown as mean ± SEM. *P < 0.05; **P < 0.01; ***P < 0.001; ****P < 0.0001. (**H**) Representative blot of mitochondrial protein content (HSP60, TIM23 and TOM20) of microparticles isolated from B16F10 cells incubated in the presence or absence of serum. The same volume of microparticle suspension was loaded in each lane, and carbonic anhydrase was used as loading control.

As reported in Figure 5A-D, B16F10 cells released a large amount of ATP-laden microparticles in the 50-250 nm size range in a P2X7R-dependent fashion. Serum starvation reduced total cell number, and therefore total amount of microparticle released was also reduced, but number of microparticles released per-cell was increased (Figure 5A-C). The ATP content of each individual microparticle was also several-fold increased in the absence of serum in both B16F10 and CT26 cells (Figure 5E and Figure S6). The P2X7R is a potent trigger of microparticle shedding 38, accordingly P2X7R silencing (Figure S7) decreased both total and per-cell microparticle shedding by at least one order of magnitude, whether incubated in the presence or absence of serum (Figure 5A-D). Microparticles are reported to contain intact mitochondria 39, thus we verified if the increased ATP content of microparticles shed in the absence of serum was due to increased mitochondrial content. Figure 5F-H shows that this is indeed the case since in the absence of serum microparticles were heavily stained by mitochondrial dyes Mitotracker Green (Figure 5F) and Mitotracker Red (Figure 5G), and contained the mitochondrial markers HSP60, TIM23 and TOM20 (Figure 5H and Figure S8). Mitotracker Red is potential-sensitive, thus its uptake shows that microparticles maintained a negative membrane potential, and therefore that trapped mitochondria were metabolically active.

**Figure 6:**
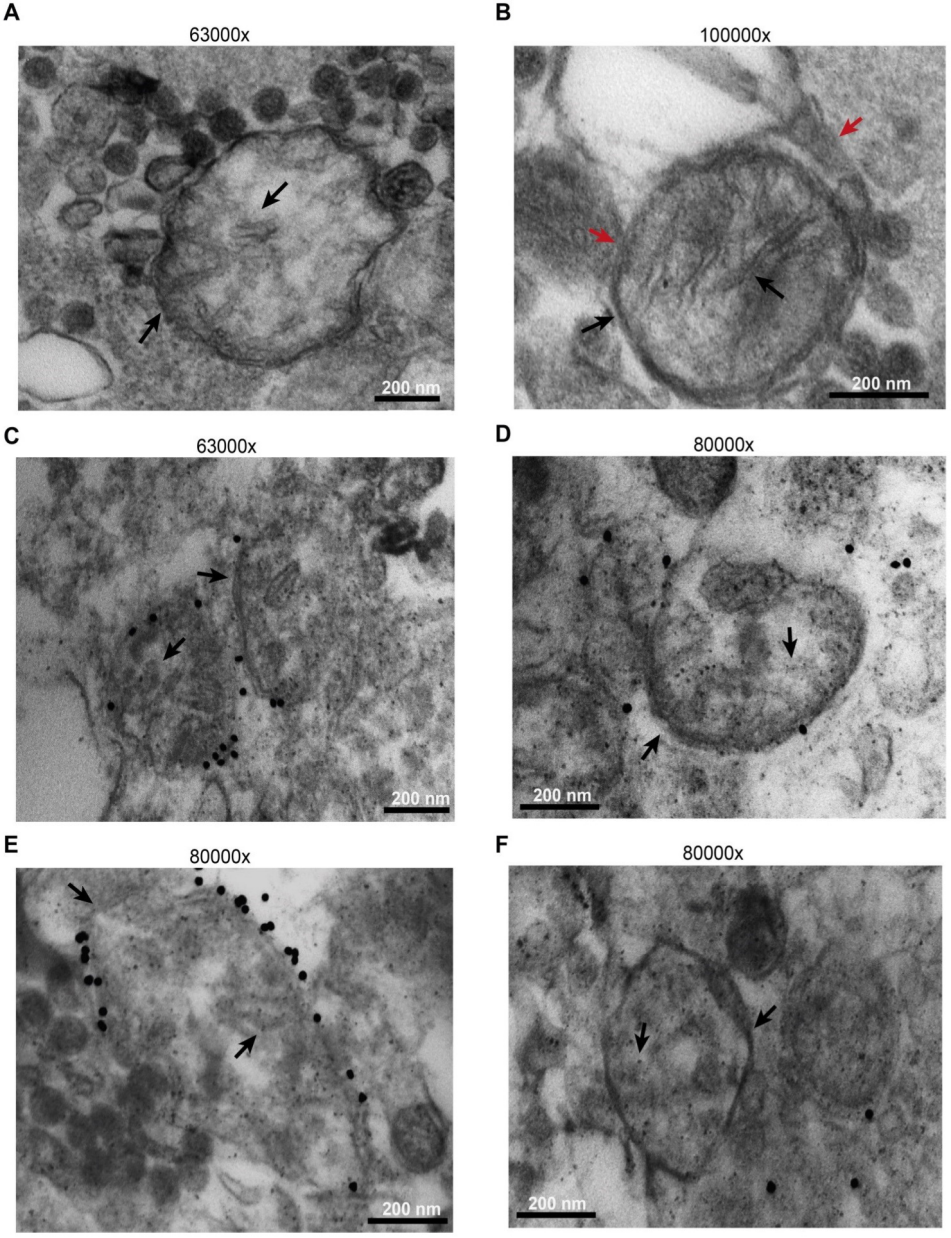
** B16F10 cells release mitochondria as both free organelles and microparticle encapsulated.** Microparticles were isolated and processed as described in Methods. Images were captured with the EM910 transmission electron microscope. (**A**) Representative picture of free mitochondria released by B16F10 cells. (**B**) Representative picture of mitochondrion trapped within a microparticle. (**C** and **E**) Immunogold staining in the presence of anti-TOM20 primary antibody. (**D** and **F**) Immunogold staining of microparticles incubated in the absence of the anti-TOM20 primary antibody (controls). Mitochondrial membrane and cristae are indicated by black arrows in all panels. Microparticle membrane in panel (**B**) is indicated by red arrow. Bars = 200 nm.

Microparticles were immunogold labelled using an anti-TOM20 primary antibody. As shown in Figure 6, while most released microparticles had a size compatible with exosomes, a few were of larger size, were bound by a double membrane and showed intraluminal cristae (black arrows). A more precise sizing of released microparticles is shown in Figure S9. Some were even enveloped by an additional membrane (Figure 6B, red arrows), suggesting entrapment within a larger vesicular structure. The outer membrane of the larger microparticles, but not the intraluminal cristae, was specifically stained by immunogold, as expected for TOM20 immunoreactivity (Figure 6C-E). Figure 6D-F shows control microparticles incubated with the gold particles in the absence of the primary antibody.

Based on electron microscopy analysis and as confirmed by Western blot (Figure S10), microparticles were highly heterogeneous since they were stained by both exosome (alix and flotillin-1) and microvesicle (calnexin) markers, and by mitochondrial markers.

**Figure 7:**
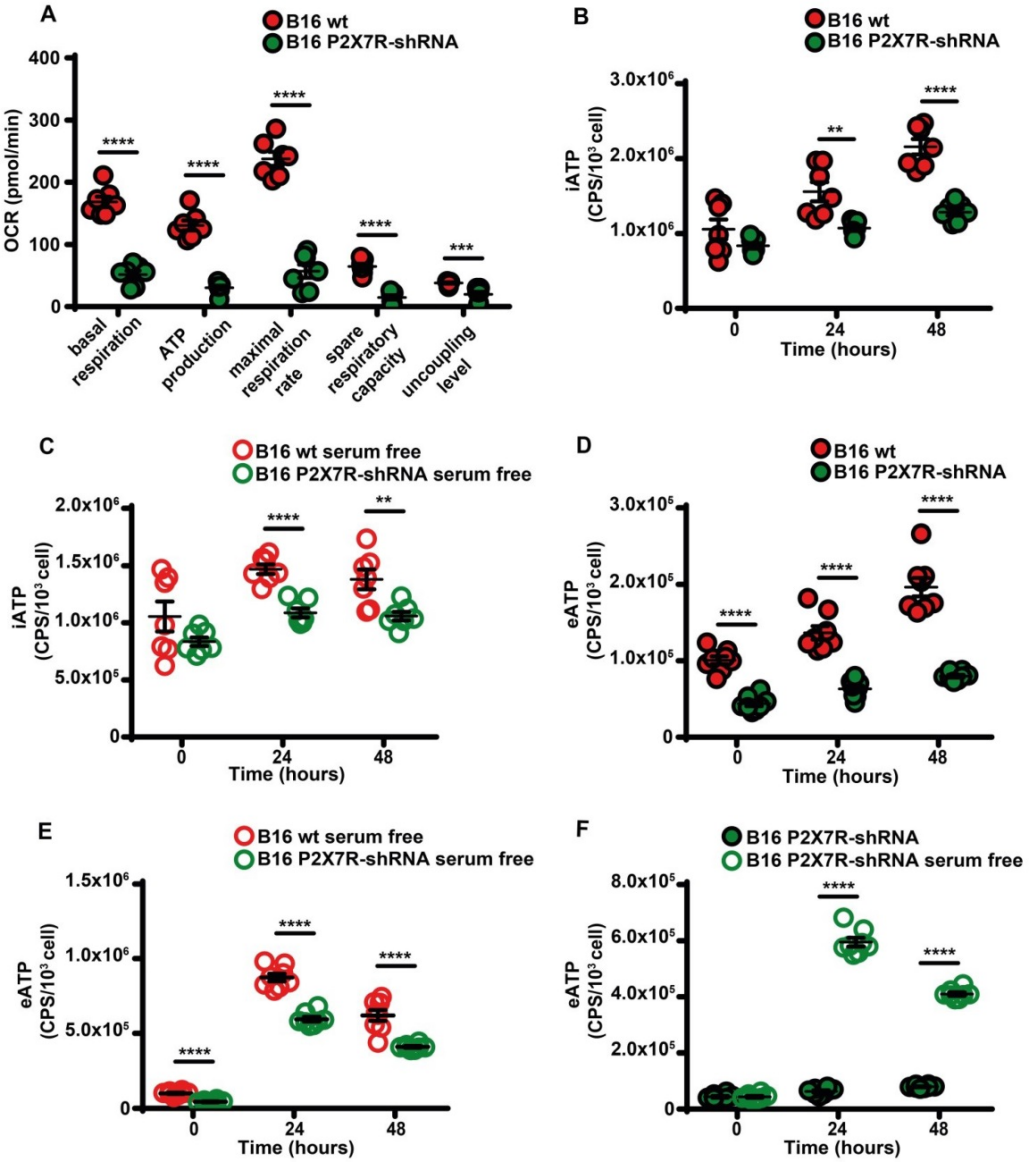
** P2X7R-silencing impairs mitochondrial energy metabolism and ATP synthesis.** (**A**) B16F10 wt (closed red circles) or P2X7R-silenced cells (B16-P2X7R-shRNA) (closed green circles) were incubated in RPMI-1640 medium and oxygen consumption rate (OCR) measured after 48 h as described in Figure 3 (n = 7). Intracellular ATP (iATP) levels were measured in B16F10 wt (closed red circles) or B16F10-P2X7R-shRNA (closed green circles) cultured in the presence (**B**) or absence (**C**) of serum. Intracellular ATP was measured with soluble luciferase after lysis with milli-Q water. CPS (counts per second) were normalized to cell content determined with crystal violet (n = 7). Extracellular ATP levels were measured in B16F10 wt (closed red circles) or B16F10-shP2X7R (closed green circles) cultured in the presence (**D**) or absence (**E**) of serum. CPS (counts per second) were normalized to cell content determined with crystal violet (n = 8). (**F**) Extracellular ATP measured with soluble luciferase in B16F10-shP2X7R cells cultured in the presence (closed green circles) or absence (open white circles) of serum. CPS (counts per second) were normalized to cell content determined with crystal violet (n = 8). Data are shown as mean ± SEM. **P < 0.01; ***P < 0.001; ****P < 0.0001.

Besides being a trigger of microparticle shedding 38, the P2X7R is also an efficient stimulant of oxidative phosphorylation 9, thus P2X7R silencing drastically reduced respiratory activity and iATP synthesis whether in the presence or absence of serum (Figure 7A-C). P2X7R silencing also reduced eATP accumulation (Figure 7D) and this effect was reverted by serum deprivation (Figure 7E-F).

### *In vivo* release of eATP by B16F10 cells is detected by tumor-infiltrating host cells

**Figure 8:**
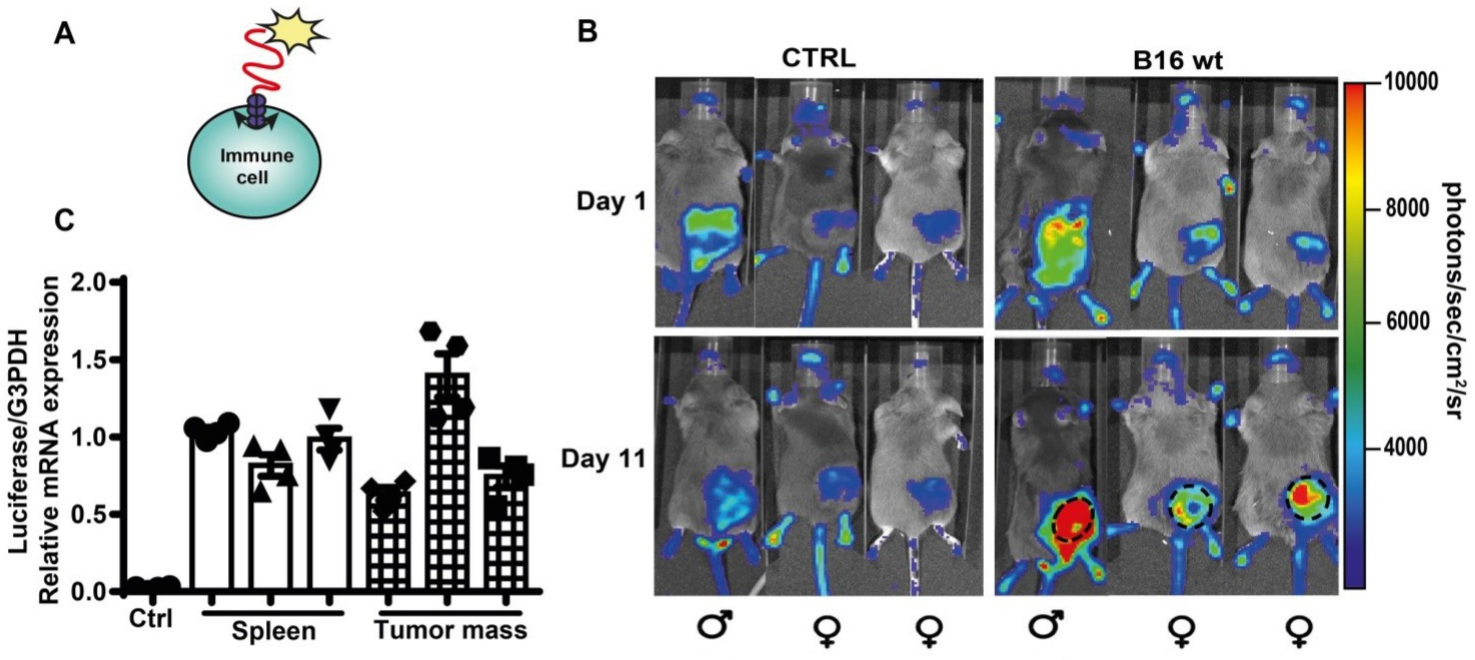
** Extracellular ATP in the TME is detected by host cell-expressed pmeLuc.** (**A**) Schematic rendition of pmeLuc expression on host immune cells showing plasma membrane localization of the probe. (**B**) Representative pictures of luminescence signal detected in pmeLuc transgenic mice. Mice on the right were shaved and inoculated with B16F10 wt cells into the right hind flank. Mice on the left (controls) were just shaved on the right hind flank. Dotted circles on the tumor-bearing mice highlight the perimeter of the tumor mass. (**C**) Relative expression by qRT-PCR of pmeLuc probe in the spleens from control and pmeLuc-TG-mice, and from inflammatory cells eluted from tumors excised from pmeLuc-TG-mice. PmeLuc expression was normalized to G3PDH (n = 3).

Accumulation of eATP into the TME has profound effects on host-tumor interaction and on the infiltrating inflammatory cells 4, 14. Previously, TME eATP 3, 12, 40 was measured with tumor-expressed pmeLuc, better suited to detect eATP close to the tumor cells, rather than in the pericellular space of host inflammatory cells. To verify whether eATP is also sensed by tumor infiltrating host cells, we used a complementary experimental model, i.e. the pmeLuc-TG-mouse engineered in our laboratory 20, 41. In this mouse virtually all cells express pmeLuc, immune cells included. The pmeLuc-TG-mouse was inoculated with B16F10-wt cells (not transfected with pmeLuc) to restrict pmeLuc expression only to host but not tumor cells. Both control and tumor-inoculated mice were shaved to minimize interference by the black fur. PmeLUC mRNA was amplified from infiltrating inflammatory cells eluted from the inoculated tumor, showing that the tumor was indeed infiltrated by host cells (Figure 8C). Luminescence emission from tumor sites was clearly visible at p.i. d 11 (Figure 8B), showing that an increase in TME eATP was detected by host cells, thus formally supporting an immunomodulatory effect of eATP in the TME.

## Discussion

ATP is one of the most common biochemical components of the TME. The elevated eATP concentration in the TME is currently being exploited for the development of highly innovative, selective and efficient anti-cancer therapies 14, 16, 17. In the TME, eATP acts as an immune-stimulant 7 or immune-suppressant, whether directly 42 or via adenosine generation 43, 44, and depending on the concentration and P2 or P1 receptor subtypes involved. In principle, both tumor cells and tumor-infiltrating inflammatory or stromal cells could release eATP in response to locally acting stimulatory factors (e.g. acidosis, hypoxia, nutrient deprivation or inflammatory cytokines) 3, 45-47, or upon activation of specific intracellular pathways (e.g. autophagy) 40. Recent findings suggest that the tumor cells themselves are the main eATP source 3, 40. This finding is paradoxical since the prevailing conditions in the TME, i.e. hypoxia and nutrient deprivation, are anticipated to impair ATP generation by reducing oxygen and substrate availability. Thus, if a relationship exists between the two ATP pools, i.e. intracellular and extracellular, eATP accumulation in the TME should be thwarted. On the contrary, eATP accumulates in the TME despite iATP depletion.

Serum-deprivation severely affected mitochondria structure and function by causing fragmentation of the mitochondrial network, inhibition of Complex I and II protein expression, and impairment of oxidative phosphorylation, which led to a gross depletion of iATP levels, yet eATP release was stimulated. Serum deprivation reduced B16F10 cell proliferation several fold but did not promote cell death, thus increased eATP release was not due to passive leakage. Several non-lytic eATP release pathways are known, i.e. ABC transporters, connexins, pannexin-1, the P2X7R itself 48, but other pathways cannot be excluded. We show here that release of ATP-laden microparticles contributed to eATP accumulation in the supernatant of B16F10 melanoma cells. Under serum deprivation, microparticle release and microparticle ATP content were both enhanced, despite cells were induced into quiescence.

Several intracellular or plasma membrane molecules, and even intracellular organelles, have been identified within microparticles 49. We found that B16F10 cells, either spontaneously or under serum deprivation, release a very heterogenous microparticle population laden with ATP and containing exosomes, microvesicles and naked mitochondria. Mitochondria are also occasionally trapped within large microparticles.

The rationale for discharging eATP into the extracellular space by cells that are starved of nutrients, and therefore in principle should preserve their iATP stores is obscure, but this is very likely the situation occurring in solid tumors which are nutrient and oxygen deprived, yet accumulate eATP into the TME 3, 12, 13, 40. Although in principle infiltrating inflammatory cells might also release eATP, our previous data suggest that this accounts only for a small fraction of eATP in the TME 13, 30, thus the majority of eATP is released from the tumor.

Since eATP accumulation into the TME heavily affects host-tumor interaction 14, 50, tightly regulated processes might have evolved to prolong its half-life. As a soluble molecule in the extracellular space, eATP half-life is very short, due to cell-associated and soluble ecto-ATPases. On the contrary, microparticle-bound ATP is likely to survive longer, and possibly reach distant destinations, since microparticles can travel long distances in the TME and even be released into circulation 51. After reaching the target cell, microparticles may either burst, thus releasing their cargo, or be taken up. Thus, microparticle-mediated ATP exchange between cells might have an important pathophysiological significance in the overall energy balance of the TME.

The P2X7R has an established role as a prime driver of microparticle release from many different cell types 38, 52, 53. Our present data show that expression of this receptor is also needed to support release of microparticles as well as of eATP from B16F10 cells. Nutrient deprivation was a potent stimulus for eATP release also in P2X7R-silenced cells, showing that in the nutrient-deprived and hypoxic TME the P2X7R might be partially dispensable for the overall eATP homeostasis.

## Conclusion

**Figure 9:**
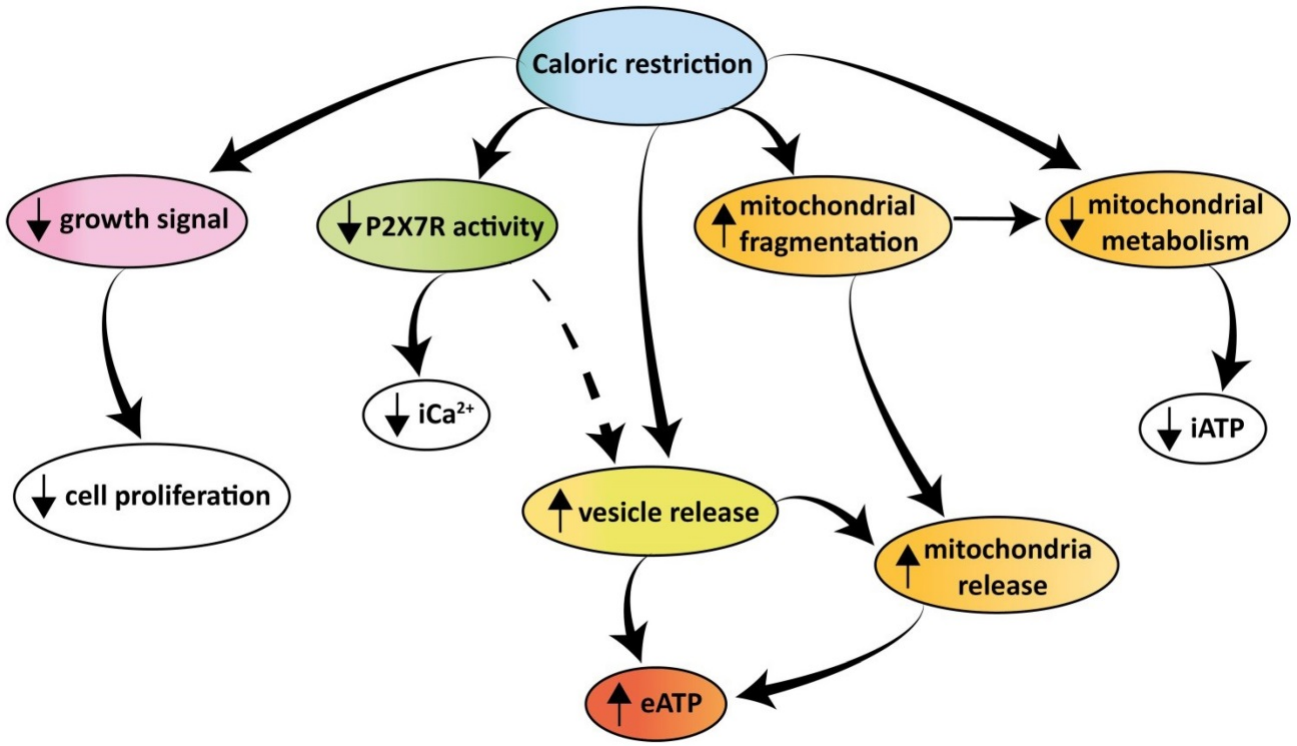
** Schematic rendition of the effects of caloric restriction on cell metabolism and eATP.** Caloric restriction inhibits growth signal generation, P2X7R function, and cell proliferation. Resting and P2X7R-stimulated intracellular Ca^2+^ level is also reduced. Mitochondria are fragmented, thus impairing energy metabolism and iATP accumulation. At the same time, caloric restriction promotes release of mitochondria-containing microparticles/microvesicles as well as of naked mitochondria. This process is accompanied by, and possibly even responsible for, release of eATP.

In conclusion our findings show that tumor cells release large amounts of eATP *in vivo* and *in vitro*. Extracellular ATP is extruded via a P2X7R-dependent process promoting the release of mitochondria-containing microparticles or even of naked mitochondria. Both microparticle and eATP release are enhanced under conditions of nutrient deprivation despite the shrinking iATP stores. The increased eATP in the TME is detected by the tumor and the infiltrating inflammatory cells, thus nutrient deprivation moulds the eATP concentration of the TME affecting both tumor and host cell responses. A schematic rendition of the multiple effects caused by caloric deprivation on cell proliferation, P2X7R function, energy metabolism, microparticle release and eATP is shown in Figure 9.

## Supplementary Material

Supplementary figures.

## Abbreviations

CRM: caloric restriction mimetics
eATP: extracellular ATP
TME: tumor microenvironment
P2X7R: P2X7 receptor
iATP: intracellular ATP
HC: hydroxycitrate
BzATP: benzoylATP
FCCP: carbonyl cyanide a-[3-(2-benzothiazolyl)6-[2-[2-[bis(carboxymethyl) amino]-5-methylphenoxy]-2-oxo-2H-1-benzopyran-7yl]-b-(carboxymethyl)-tetrapotassium salt
EVs: extracellular vesicles
shRNA: short hairpin RNA
pmeLuc-Tg-mouse: transgenic pmeLuc mouse
i.p.: intra peritoneal
p.i.: post injection
LDH: lactic dehydrogenase
OCR: oxygen consumption rate
ECAR: extracellular acidification rate
