# Supplementary material for: Extracellular ATP is increased by release of ATP-loaded microparticles triggered by nutrient deprivation
Source: Theranostics. 2022 Jan 1;12(2):859–74. doi: 10.7150/thno.66274 (PMC8692914; doi:10.7150/thno.66274)
Supplement: Supplementary file 1 — Supplementary figures. [file thnov12p0859s1.pdf]

Supplementary Figures

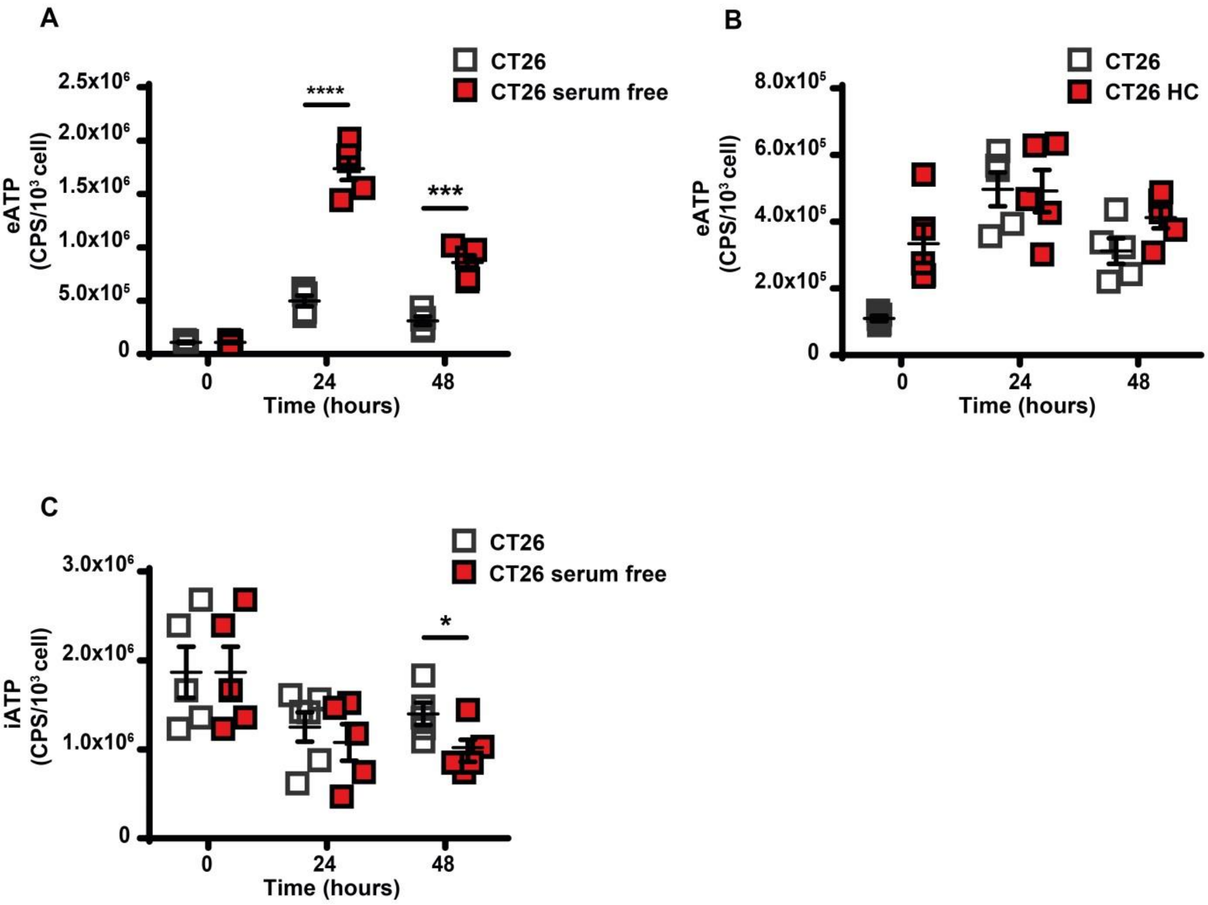

Figure S1. Serum starvation and hydroxycitrate promote ATP release from CT26 colon carcinoma cells *in vitro*.

(**A**) Extracellular ATP levels in CT26 cells (5×10^3) cultured in RPMI-1640 medium in the presence (open squares) or absence (closed red squares) of serum. (**B**) eATP levels measured in CT26 cells incubated in the presence (closed red squares) or absence (open squares) of 1 mM HC. eATP was measured with soluble luciferase. CPS (counts per second) were normalized to cell content measured with crystal violet. (**C**) CT26 cells (5×10^3) were incubated in RPMI-1640 medium in the presence (open squares) or absence (red closed squares) of serum, at the indicated time were lysed and intracellular ATP (iATP) measured by luciferase assay (n = 5). Data are shown as mean ± SEM. *P < 0.05; ***P < 0.001; ****P < 0.0001.

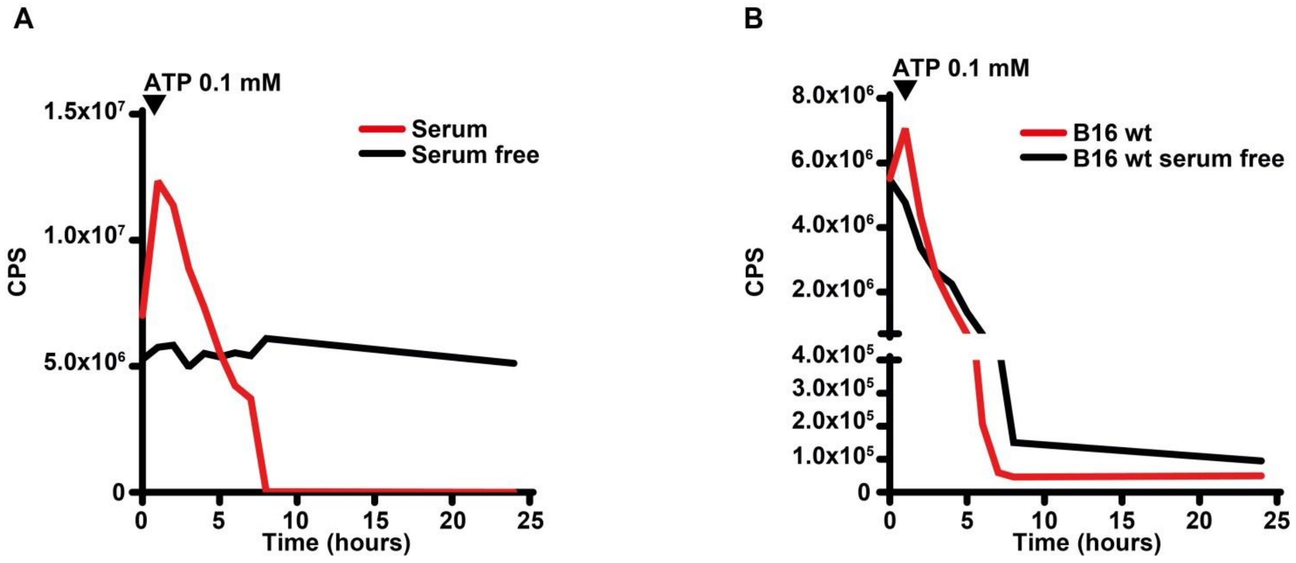

Figure S2. Rate of hydrolysis of added ATP in cell-free RPMI medium (a) or in RPMI medium in the presence of B16F10 monolayer (b).

(**A**) ATP hydrolysis was measured in serum-containing (red line) or serum-free (black line) RPMI-1640 medium, or (**B**) in B16F10 monolayers (100.000 cells /well) incubated in the presence (red line) or absence (black line) of serum. Luminescence increase observed soon after the addition of ATP to serum-containing medium is due to ATP-triggered release of ATP from extracellular vesicles contained in the serum.

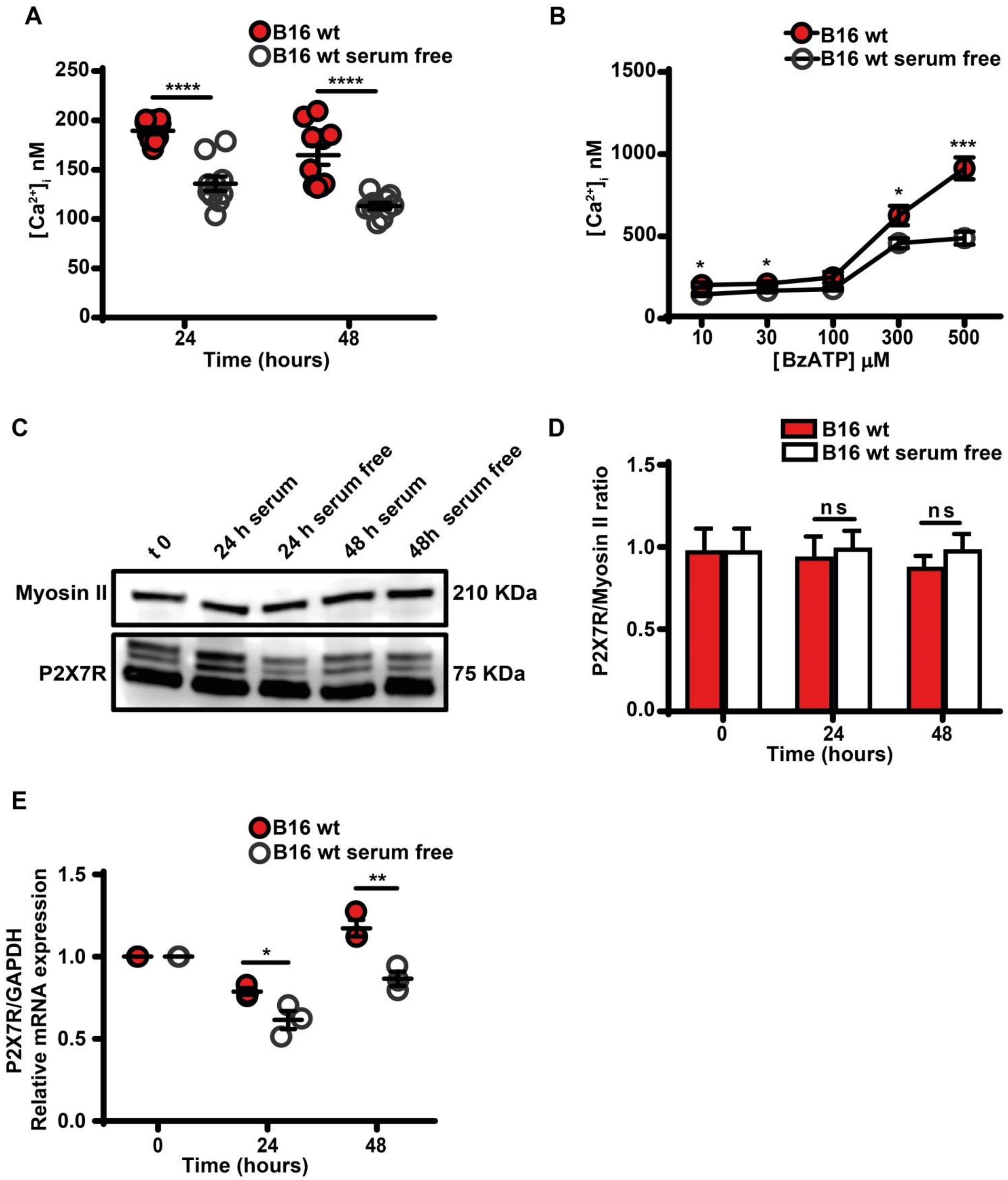

Figure S3. Serum deprivation impairs P2X7R-dependent responses.

(**A**) Basal cytosolic Ca^{2+} levels measured with Fura 2/AM in B16F10 cells (2×10^6) incubated in RPMI-1640 medium in the presence (closed red circles) or absence (open circles) of serum (n = 10).

(**B**) Dose-dependency of BzATP-stimulated cytosolic Ca^{2+} changes in B16F10 cells (2×10^6) cultured in RPMI-1640 medium in the presence (closed red circles) or absence (open circles) of serum (n = 6). (**C**) Western blot analysis and (**D**) densitometry (n = 4) of P2X7 protein expression in serum- supplemented or serum-starved B16F10 cells (1×10^5) at different incubation time points. Fifteen micrograms of protein was loaded in each lane. (**E**) Relative expression of the P2X7 mRNA in B16F10 cells cultured in the presence (closed red circles) or absence (open circles) of serum measured by qRT-PCR. Data were normalized to GAPDH expression as endogenous control (n = 3). Data are shown as mean ± SEM. *P < 0.05; **P < 0.01; ***P < 0.001; ****P < 0.0001

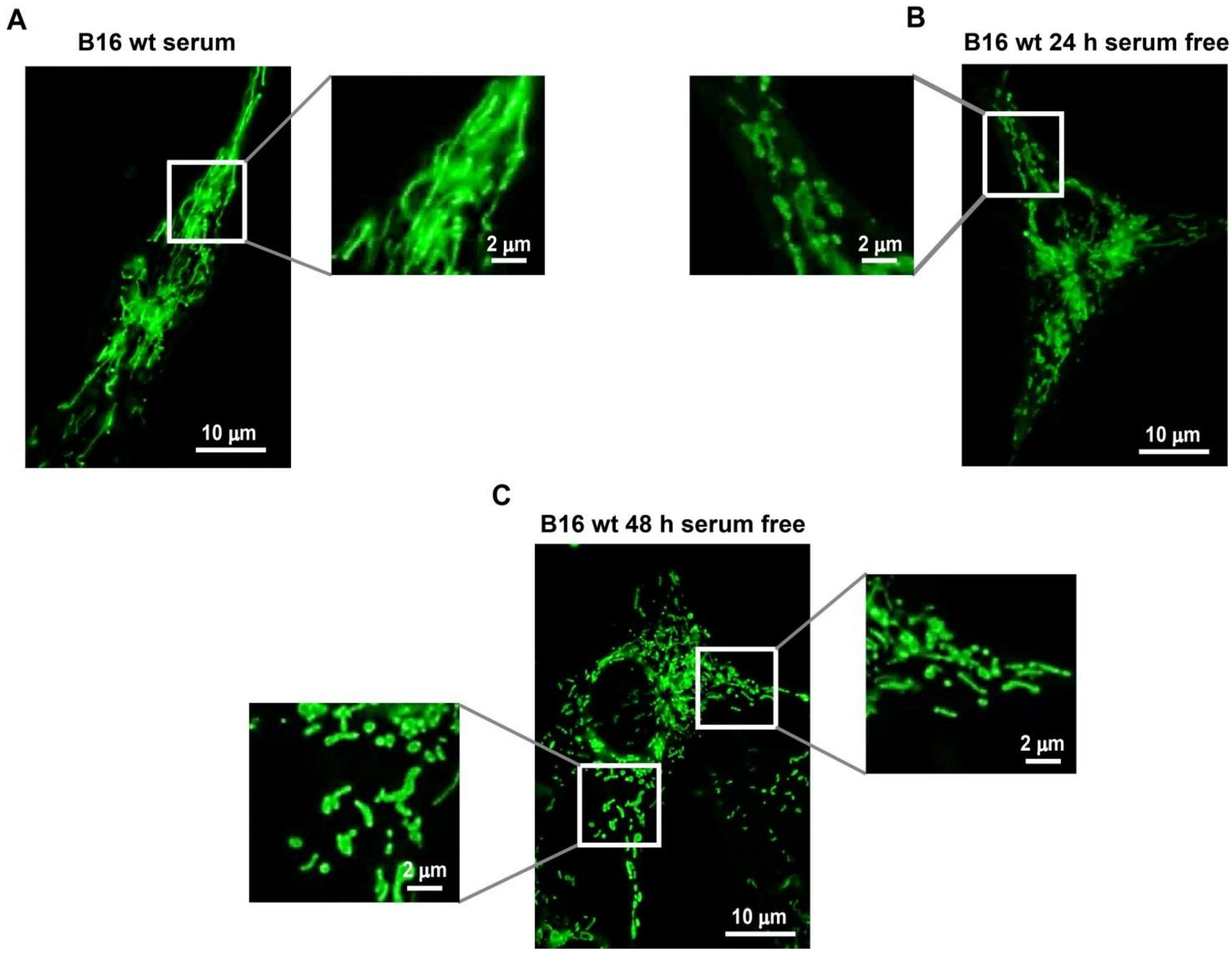

Figure S4. Serum deprivation causes fragmentation of the mitochondrial network.

B16F10 wt cells were incubated in the presence (**A**) or absence (**B** and **C**) of serum and loaded with Mitotracker Green (200 nM). Images were captured with an inverted Nikon Eclipse TE300 microscope equipped with a 100×/05 -1.3 NA oil Iris objective and analyzed using the Metamorph software. Bars = 10 µm (2 µm in the insets).

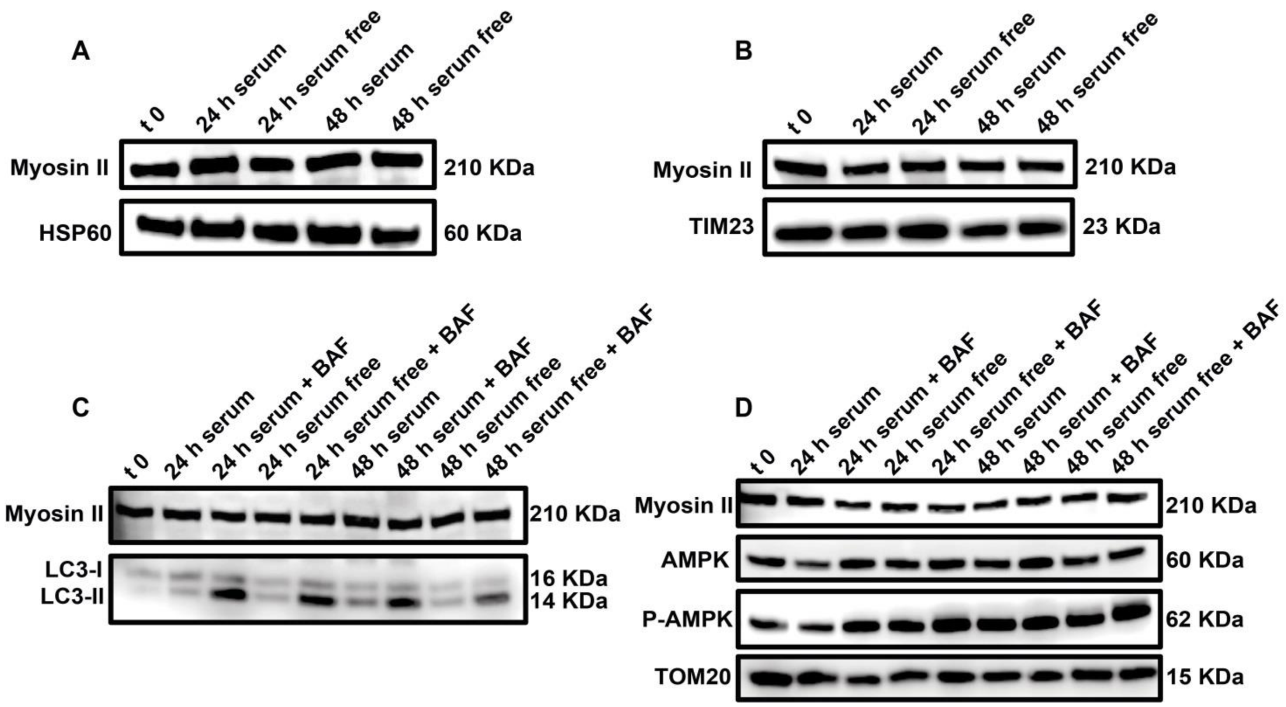

Figure S5. Serum deprivation does not activate mitophagy or AMPK in B16F10 cells.

B16F10 wt cells were cultured in the presence or absence of serum for 24 or 48 h and analyzed for Hsp60 (**A**), TIM23 (**B**), LC3-I and LC3-II (**C**) or AMPK, phospho-AMPK (P-AMPK) and TOM20 (**D**) expression. Bafilomycin A1 (BAF**,** 100 nM) was used to inhibit the autophagic flux.

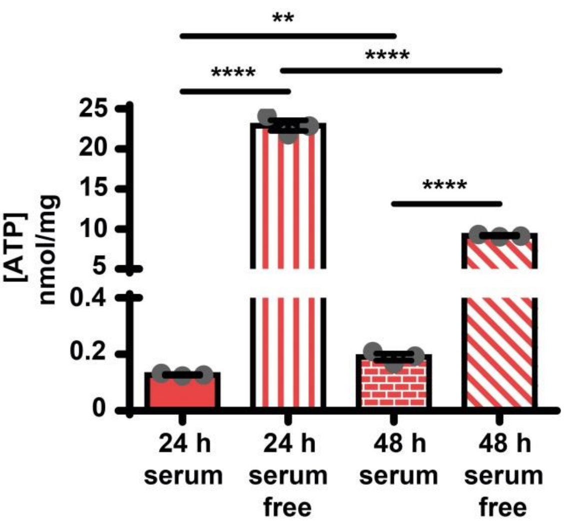

Figure S6 ATP content of CT26-derived microparticles

Microparticles were isolated from CT26 cells incubated for 24 or 48 h in the presence or absence of serum as described in Methods. Microparticles were lysed and ATP content measured with the luciferase assay (n = 3). Data are shown as mean ± SEM. **P < 0.01; ***P < 0.001; ****P < 0.0001.

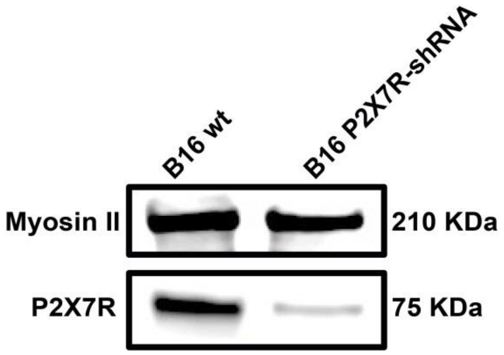

Figure S7. P2X7 receptor-silencing in B16F10 cells.

Western blot of the P2X7R in B16F10 wt and P2X7R-silenced (B16 P2X7R-shRNA) cells carried out as described in Methods.

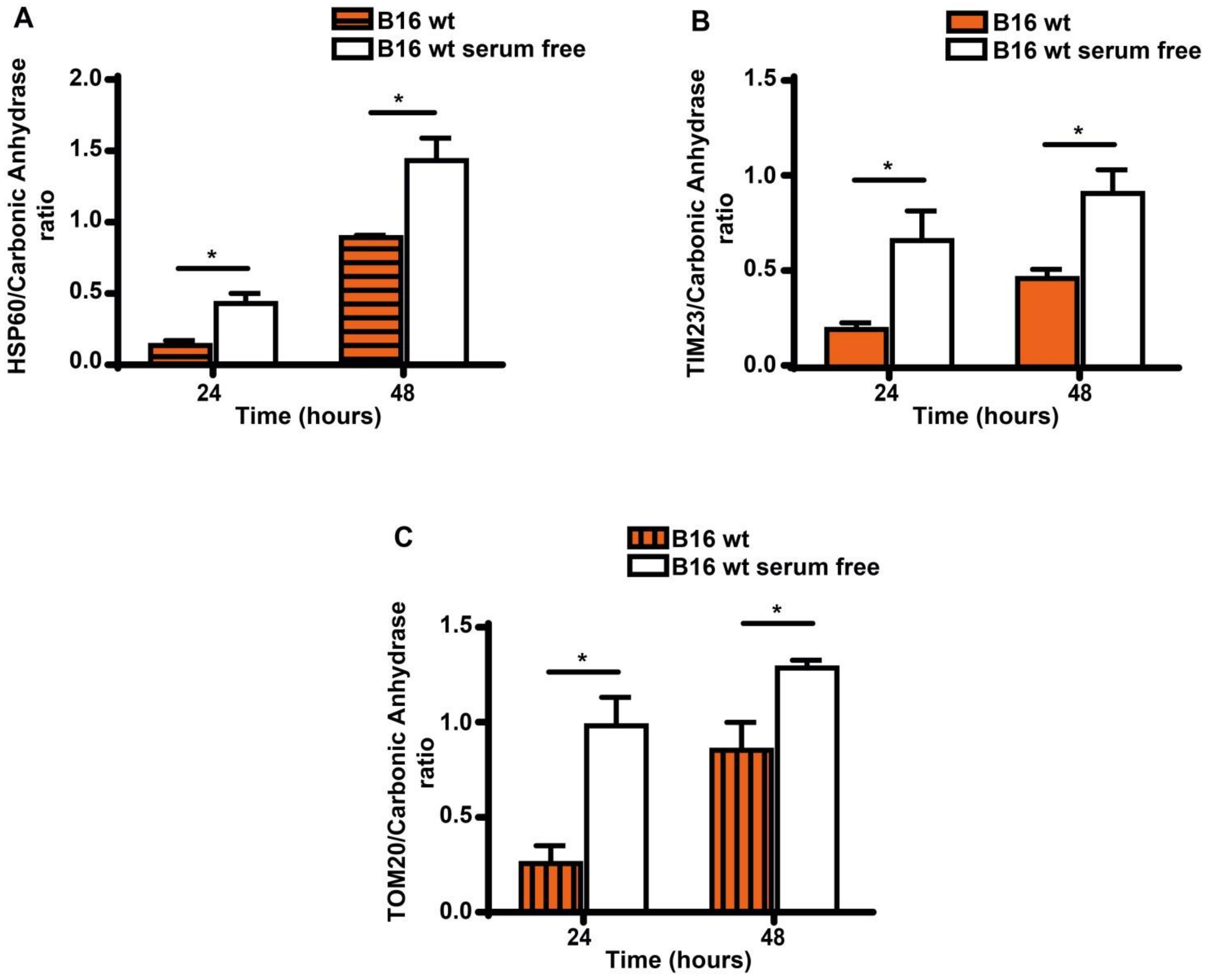

Figure S8. Microparticles released under serum-free conditions have a higher content of mitochondrial markers.

Densitometric analysis of Western blot shown in Fig. 5H. HSP60 (**A**), TIM23 (**B**) and TOMM20 (**C**) expression in microparticles isolated from B16F10 cells incubated in the presence or absence of serum. Data are means ± SEM of independent determinations from three separate Western blots. *P < 0.5

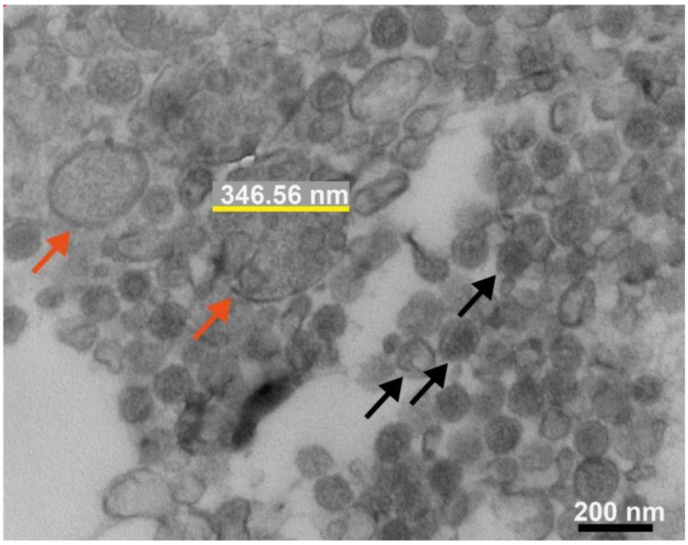

Figure S9 Microparticle sizing.

Microparticles were isolated and processed for electron microscopy as described in Materials and Methods. Red arrows, microvesicles; black arrows, exosomes.

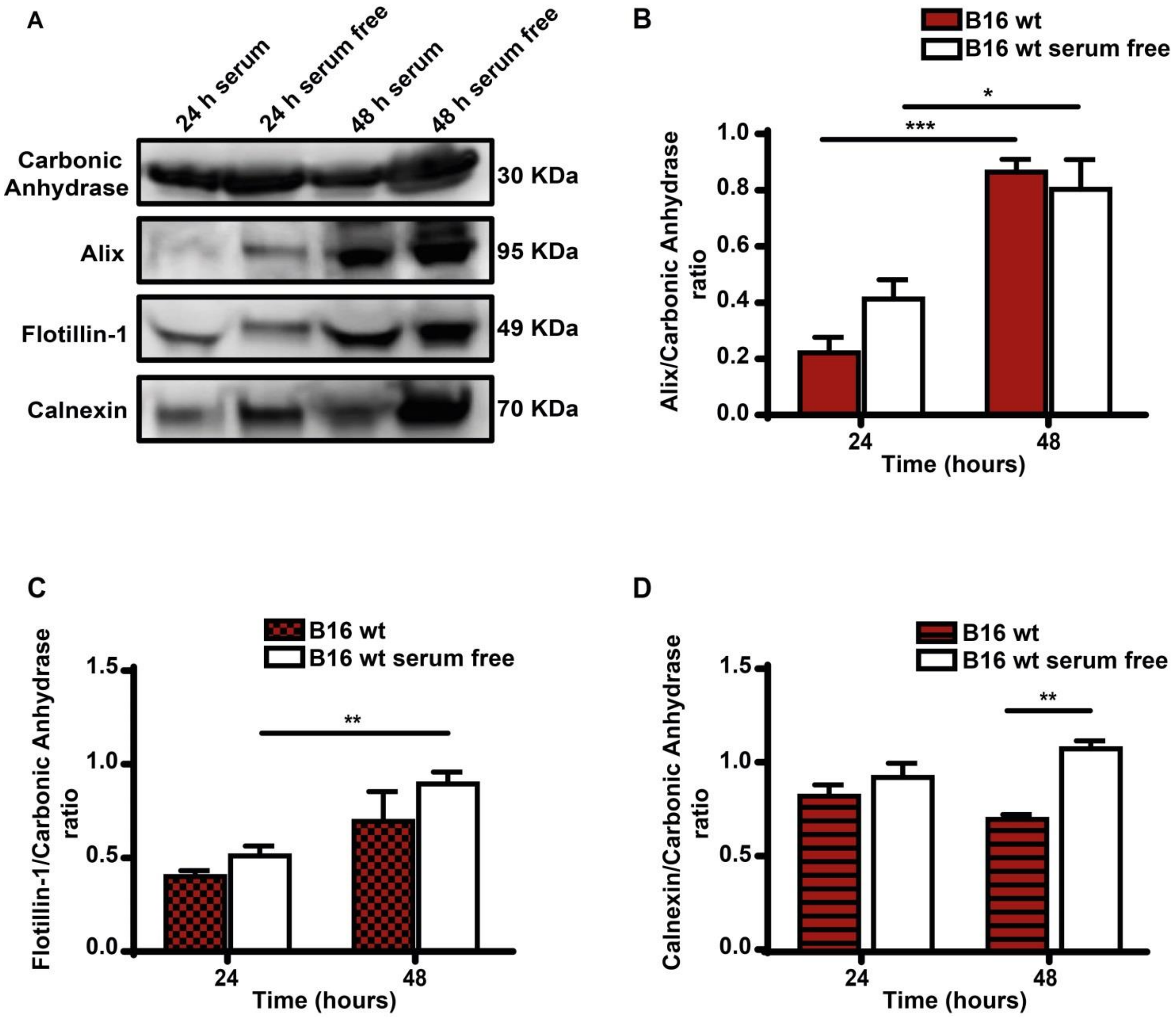

Figure S10. Microparticles released in the presence or absence of serum contain both exosomes and microvesicles.

Specific exosome (alix and flotillin-1) and microvesicle (calnexin) markers in microparticles released from B16F10 cells incubated in the presence or absence of serum for 24 or 48 h were identified by Westen blot analysis. (**A)** Representative Western blot. Carbonic anhydrase was used as loading control. Densitometry of alix (**B**), flotillin-1 (**C**) and calnexin (**D**). Data are means ± SEM from the analysis of three independent Western blots from three separate preparations. *P < 0.5; **P < 0.05; ***P < 0.005.
